# Multi-Class Cost-Constrained Random Coding for Correlated Sources over the Multiple-Access Channel

**DOI:** 10.3390/e23050569

**Published:** 2021-05-03

**Authors:** Arezou Rezazadeh, Josep Font-Segura, Alfonso Martinez, Albert Guillén i Fàbregas

**Affiliations:** 1 Department of Electrical Engineering, Chalmers University of Technology, SE-412 96 Gothenburg, Sweden; 2 Department of Information and Communication Technologies, Universitat Pompeu Fabra, 08018 Barcelona, Spain; josep.font@upf.edu (J.F.-S.); alfonso.martinez@upf.edu (A.M.); albert.guillen@upf.edu (A.G.i.F.); 3 Institució Catalana de Recerca i Estudis Avançats, 08010 Barcelona, Spain; 4 Department of Engineering, University of Cambridge, Cambridge CB2 1PZ, UK

**Keywords:** multiple access channel, correlated sources, random coding, error exponents

## Abstract

This paper studies a generalized version of multi-class cost-constrained random-coding ensemble with multiple auxiliary costs for the transmission of *N* correlated sources over an *N*-user multiple-access channel. For each user, the set of messages is partitioned into classes and codebooks are generated according to a distribution depending on the class index of the source message and under the constraint that the codewords satisfy a set of cost functions. Proper choices of the cost functions recover different coding schemes including message-dependent and message-independent versions of independent and identically distributed, independent conditionally distributed, constant-composition and conditional constant composition ensembles. The transmissibility region of the scheme is related to the Cover-El Gamal-Salehi region. A related family of correlated-source Gallager source exponent functions is also studied. The achievable exponents are compared for correlated and independent sources, both numerically and analytically.

## 1. Introduction

In information theory, the fundamental problem of communication over a channel is studied from two complementary perspectives. First, one characterizes the transmissibility conditions, namely the circumstances under which the error probability asymptically vanishes as the blocklength goes to infinity. Second, one describes by means of error exponents the speed at which this error probability vanishes; the larger the exponent, the faster the error probability tends to zero. Since finding an exact expression for error probability is very difficult, a large body of work has investigated upper and lower bounds on the average error probability, or equivalently lower and upper bounds for the error exponent. In point-to-point, that is, single-user communication, using separate source-channel random coding [1,2], possibly with expurgation [1] (Eq. 5.7.10), yields lower bounds on the error exponent. In contrast, finding an upper bound to the error exponent satisfied by every code is more challenging. Generally, the hypothesis-testing method [3] is employed to derive upper bounds for the error exponent. Two well-known upper bounds to the error exponent are the sphere-packing exponent [4] and the minimum-distance exponent [5]. In fact, for rates greater than critical rate [1] (Sec. 5.6), the random-coding and sphere-packing bounds coincide with each other, while the expurgated and minimum-distance bounds coincide at rate zero.

For point-to-point communication, it was shown in ref. [1] (Prob. 5.16) that joint source-channel coding leads in general to a larger exponent than separate source-channel coding. Additionally, using codewords with a composition dependent on the source message leads to a larger exponent than the case where codewords are drawn according to a fixed product distribution [6,7]. Moreover, a scheme where source messages are assigned to disjoint classes and encoded by codes that depend on the class index, attains the sphere-packing exponent in those cases where it is tight [8].

Many works have been devoted to studying the transmissibility and the error exponent for a two-user multiple-access channel (MAC) [9,10,11]. Separate source-channel coding for the MAC with independent sources was studied in refs. [9,12]. In ref. [13], a universal exponent for the MAC was derived by considering separate source-channel coding. In ref. [14], a transmissible region is derived for the MAC under mismatched decoding, where the decoding rule is fixed and possibly suboptimal. In ref. [15], it was shown that using structure coding can improve the error exponent of the MAC. The maximum-error-probability criterion and the impact of feedback for the MAC were studied in ref. [16]. By considering separate source-channel coding, lower and upper bounds for the error exponent of the MAC were respectively obtained in refs. [17,18]. For the MAC with independent sources, the idea of exploting the dependency between messages and codewords was studied in ref. [19]. In ref. [20], an achievable exponent for the MAC with independent sources was given in the dual domain, that is, as a lower dimensional problem over parameters in terms of Gallager functions. For the MAC with correlated sources, it was shown in ref. [11] that considering statistical dependency between messages and codewords for the MAC with correlated sources leads to a larger transmissible region. However, an example presented in ref. [21] shows that one can reliably transmit information through the MAC without satisfying the reliable transmission obtained in ref. [11]. In another line of work, superposition coding with Gacs Körner Witsenhausen (GKW) common part is used in ref. [22] to to describe the sufficient conditions lossless recoverability.

In contrast to single-user communication, the problem of reliable transmission of two correlated sources has not been solved yet and just the sufficient conditions of a reliable transmission has been derived. In ref. [23], by applying coding techniques, a new set of sufficient conditions were proposed. Moreover, in ref. [24] new sufficient conditions for the three-user MAC with correlated sources were studied. In ref. [25], an achievable exponent derived was presented in the primal domain, that is, as a multi-dimensional optimization problem over distributions that is generally difficult to analyze.

In this paper, we examine how statistical dependency between the messages and codewords improves the exponent, as well as its impact on the transmissibility region. In view of refs. [1] (Ch. 7) and [26], we study a generalized message-dependent cost-constrained random-coding ensemble with multiple cost functions. By choosing the proper cost functions, the multi-class cost-constrained ensemble subsumes multiple ensembles previously considered in the literature and recovers the transmissibility region in ref. [11].

The paper is organized as follows—in Section 2, we present the problem of transmission of *N* correlated sources over an *N*-input discrete memoryless multiple-access channel and provide the key definitions of error probability, transmissibility, random-coding ensemble, and achievable exponent. In Section 3, we review the existing random-coding ensembles, define a novel generalized multi-class cost-constraint ensemble and characterize its achievable exponent. In the discussion Section 4, we characterize the transmissibility region for our error exponent, relate the exponent to standard Gallager source and channel functions, and provide numerical results and formulas that allow us to rank the exponents attained by the various standard random-coding ensembles.

## 2. Problem Formulation

We study the simultaneous transmission of *N* correlated, discrete, memoryless sources over a channel; users are indexed by ν∈N={1,2,…,N}. The source messages $u_{\nu }$ of user ν have *n* symbols drawn from the alphabet $U_{\nu }$. We denote by $u_{\sigma }$ the ordered vector of source messages for all users in a set $\sigma \subset 2^{N}$, i.e., a subset of the set of all user indices, and similarly by $U_{\sigma }$ the Cartesian product of the source alphabets in the set σ. When σ=N, $u_{N}$ and $U_{N}$ denote the ordered vector of source messages for all users and the Cartesian product of the all source alphabets respectively. The sources are memoryless and are characterized by the joint probability distribution $P_{N}$

(1) $$\begin{array}{c} P_{N}\left(u_{N}\right)=\prod_{t=1}^{n}P_{N}\left(u_{N,t}\right), \end{array}$$

and by the symbol joint probability distribution $P_{N}$. The source message and symbol marginal distributions of user ν∈N are denoted by $P_{\nu }$ and $P_{\nu }$ respectively. Assuming that the sources are independent, the marginal distributions induce new joint (mismatched) probability distributions of sets of users $\sigma \subset 2^{N}$. The induced independent-message and -symbol probabilities, denoted by $P_{\sigma }^{\mathrm{ind}}$ and $P_{\sigma }^{\mathrm{ind}}$, are given by

(2) $$\begin{array}{c} P_{\sigma }^{\mathrm{ind}}\left(u_{\sigma }\right)=\prod_{\nu \in \sigma }P_{\nu }\left(u_{\nu }\right), \end{array}$$

and similarly for $P_{\sigma }^{\mathrm{ind}}$.

Each user ν has an encoder that maps, without cooperation with the other users, the source message $u_{\nu }$ onto a codeword $x_{\nu }\left(u_{\nu }\right)$ also of length *n* and with symbols drawn from the alphabet $X_{\nu }$. We denote the codebook of user ν by $C_{n}^{\nu }$. We denote by $x_{\sigma }\in X_{\sigma }^{n}$ the vector of codewords for all users in a set $\sigma \subset 2^{N}$. Both terminals simultaneously send these codewords over a discrete memoryless multiple access channel with output alphabet Y. The symbolwise transition probability is denoted by *W*, and the channel is characterized by a conditional probability distribution

(3) $$\begin{array}{c} W\left(y|x_{N}\right)=\prod_{t=1}^{n}W\left(y_{t}|x_{N,t}\right), \end{array}$$

where y is the received sequence of length *n*.

Based on y, a joint decoder estimates all transmitted source messages $u_{N}$ according to the maximum a posteriori criterion:

(4) $$\begin{array}{c} {\hat{u}}_{N}=\underset{u_{N}\in U_{N}^{n}}{arg max}P_{N}\left(u_{N}\right)W\left(y\;|\;x_{N}\left(u_{N}\right)\right), \end{array}$$

where $U_{N}^{n}$ denotes the set of all possible source messages $u_{N}$. An error occurs if the decoded messages ${\hat{u}}_{N}$ differ from the transmitted $u_{N}$; we refer to ${\hat{u}}_{N}\neq u_{N}$ as an error event. The error probability for a given set of codebooks, $P_{e}\left(C_{n}^{N}\right)$, is thus given by

(5) $$\begin{array}{c} P_{e}\left(C_{n}^{N}\right)≜\mathrm{Pr}\left\{{\hat{U}}_{N}\neq U_{N}\right\}. \end{array}$$

In our analysis, it will prove convenient to split the error event into $2^{N}-1$ distinct types of error events indexed by the non-empty subsets in the power set of the user indices $2^{N}\backslash \emptyset$, for example, τ∈{{1},{2},{1,2}} for N=2. More precisely, the error event of type τ corresponds to the conditions ${\hat{u}}_{\nu }\neq u_{\nu }$ for all ν∈τ and ${\hat{u}}_{\nu }=u_{\nu }$ for all $\nu \in \tau^{c}$, where $\tau^{c}$ is the complement of τ in the power set of the user indices.

We are interested in the asymptotics of the error probability for sufficiently large *n*, namely whether the error probability vanishes and how fast this probability tends to zero as it vanishes. The sources $U_{N}$ are said to be transmissible over the channel if there exists a sequence of codebooks $C_{n}^{N}$ such that $\lim_{n\to \infty }P_{e}\left(C_{n}^{N}\right)=0$. To characterize the speed at which the error probability vanishes, we use the notion of exponent. An exponent E is said to be achievable if there exists a sequence of codebooks such that

(6) $$\begin{array}{c} \underset{n\to \infty }{lim inf}-\frac{1}{n}\log P_{e}\left(C_{n}^{N}\right)\geq E. \end{array}$$

Source transmissibility and error-exponent achievability are typically studied by means of random coding. With random coding, one generates and studies sequences of ensembles of codebooks whose codewords are randomly drawn from a distribution $Q_{\nu }\left(x_{\nu }|u_{\nu }\right)$ independently for each user; as indicated by the notation, this distribution may possibly depend on the source message $u_{\nu }$. The random-coding probability distribution for the channel input $Q_{N}\left(x_{N}|u_{N}\right)$ combined for all users is given by

(7) $$\begin{array}{c} Q_{N}\left(x_{N}|u_{N}\right)=\prod_{\nu \in N}Q_{\nu }\left(x_{\nu }|u_{\nu }\right). \end{array}$$

The use of random coding allows us to study how the error probability averaged over the ensemble, denoted by ${\bar{P}}_{e}$ vanishes as *n* grows. More importantly, it shows the existence of good codes in the ensemble such that their error probability vanishes. For the point-to-point and the multiple-access channels, a number of such random-coding ensembles have been studied in the literature, as reviewed in the following section, where we also present a multi-class cost-constrained ensemble subsuming all these ensembles and characterize the achievable exponent and transmissibility region of this ensemble.

### Summary of Notation Used in the Paper

Sets are usually denoted by calligraphic upper case letter, e.g., X, and the *n*-Cartesian product set of X is denoted by $X^{n}$. The cardinality of a set such as X is denoted by |X|. The indicator function representing an error event or that an element *x* belongs to a set X is denoted by 1{x∈X}.

The number of users is denoted by *N* and user indices are typically represented by ν. The set of all users is denoted by N. The power set of all subsets of N is denoted by $2^{N}$ and the complement of a subset $\sigma \subset 2^{N}$ is denoted by $\sigma^{c}$; sets in the power set of users are denoted that by Greek letters, for example, τ and σ. The number of source-message classes and of cost functions for user ν are respectively denoted by $K_{\nu }$ and $L_{\nu }$; the sets of such classes are functions are respectively denoted by $K_{\nu }$ and $L_{\nu }$. Indices for source classes and cost functions are typically denoted by $i_{\nu }$ and $\ell_{\nu }$ respectively.

Subscripts and superscripts in a quantity *A* may represent sets of user indices σ. Depending on the context, the quantity represents a list or a suitable product of variables for all elements in the set σ. For instance, for σ={1,2}, $A^{\sigma }=\left(A^{1},A^{2}\right)$ or $A_{\sigma }=\left(A_{1},A_{2}\right)$. If the quantity is a probability distribution, its value for σ represents the probability distribution of the sequence, for example, $Q_{\sigma }^{i_{\sigma }}\left(x_{\sigma }\right)=\prod_{\nu \in \sigma }Q_{\nu }^{i_{\nu }}\left(x_{\nu }\right)$. If the quantity is a set, its value for σ is the Cartesian product, for example, $U_{\sigma }=U_{1}\times U_{2}$ for σ={1,2}. If σ=∅, then $A_{\sigma }=A^{\sigma }=0$. If σ is a singleton, for example, σ={2}, we simply write $A_{2}$ or $A^{2}$. We denote the operation that merges and sorts two lists $A_{\sigma_{1}}$ and $A_{\sigma_{2}}$ with $\sigma_{1}\cap \sigma_{2}=\emptyset$ into an ordered list containing all users in the union $\sigma_{1}\cup \sigma_{2}$ by $\left[A_{\sigma_{1}},A_{\sigma_{2}}\right]$. For sets of user indices, we denote such merging operation by $\left[\sigma_{1},\sigma_{2}\right]$ and we have $[\sigma ,\sigma^{c}]=N$.

Scalar random variables are denoted by capital letters, for example, *X* and lowercase letters represent a particular realisation, for example, x∈X. Capital bold letter denotes random vectors or sequences, for example, X, while small bold letter $x\in X^{n}$ denote deterministic vectors or sequences. Probability distributions for vectors or sequences, typically of length *n*, (resp. for symbols) are represented by text-style letters, for example, P, Q, W (resp. math-style letters, for example, *P*, *Q*, *W*). Sequences symbols are usually affixed a subscript to indicate a user index; the *t*-th symbol in the sequence $x_{\nu }$ is denoted by $x_{\nu ,t}$.

The source-symbol distribution for user ν is denoted by $P_{\nu }\left(u_{\nu }\right)$. The joint distribution for users σ is denoted by $P_{\sigma }\left(u_{\sigma }\right)$; the joint distribution, computed as if the sources were independent, is denoted by $P_{\sigma }^{\mathrm{ind}}\left(u_{\sigma }\right)$. The conditional source distribution for users $\sigma_{1}$ given another set $\sigma_{2}$ is denoted by $P_{\sigma_{1}|\sigma_{2}}\left(u_{\sigma_{1}}|u_{\sigma_{2}}\right)$. Vector or sequence distributions are defined analogously with *P* replaced by P. Channel input distributions are denoted by $Q_{\nu }\left(x_{\nu }\right)$, $Q_{\nu }^{i_{\nu }}\left(x_{\nu }\right)$, or $Q_{\nu ,u_{\nu }}^{i_{\nu }}\left(x_{\nu }\right)$, where $i_{\nu }$ denotes the index of the class source message and $Q_{\nu ,u_{\nu }}\left(x_{\nu }\right)$ is a shorthand for the conditional distribution $Q_{\nu }\left(x_{\nu }|u_{\nu }\right)$. Cost functions are similarly denoted by $a_{\nu }\left(x_{\nu }\right)$, $a_{\nu }^{i_{\nu }}\left(x_{\nu }\right)$, or $a_{\nu ,u_{\nu }}^{i_{\nu }}\left(x_{\nu }\right)$. Vector or sequence distributions are defined analogously with *Q* or *a* respectively replaced by Q or a. The conditional distribution for the channel output symbol (resp. sequence) is denoted by $W(y|x_{N})$ (resp. $W(y|x_{N})$).

## 3. Multi-Class Cost-Constrained Ensemble with Statistical Dependency

### 3.1. Review of Random-Coding Ensembles

The simplest and oldest random-coding ensemble is the independent, identically distributed (*iid*) [1,12,17,27], where the symbols $x_{\nu ,t}$ in all codewords $x_{\nu }$ of a given user ν are generated independently according to the same input distributions $Q_{\nu }\left(x_{\nu ,t}\right)$ for all source messages $u_{\nu }$. Throughout the paper, we shall identify ensembles by hyphenated acronyms, where the first part indicates the possible dependence of the codeword on the source message and the second part describes the generation of symbols in a codeword. This first ensemble is thus the message-independent iid (*mi-iid*) ensemble, since codewords have the same distribution for all source messages and symbols are independent of each other and independent of the source message symbols too. For the mi-iid ensemble, the random-coding distribution is given by

(8) $$\begin{array}{c} Q_{\nu }^{\mathrm{mi}-\mathrm{iid}}\left(x_{\nu }|u_{\nu }\right)=\prod_{t=1}^{n}Q_{\nu }\left(x_{\nu ,t}\right). \end{array}$$

In the message-independent, independent-conditionally-distributed (*mi-icd*) ensemble, the codewords $x_{\nu }$ of user ν are generated identically for all source messages $u_{\nu }$, independently of the full message $u_{\nu }$, and with symbols according to a set of $|\;U_{\nu }|$ conditional probability distributions $Q_{\nu ,u_{\nu }}\left(x_{\nu }\right)≜Q_{\nu }\left(x_{\nu }|u_{\nu }\right)$. To this end, let $I_{u_{\nu }}\left(u_{\nu }\right)$ denote the set of positions where the symbol $u_{\nu }\in U$ appears in the sequence $u_{\nu }$, namely

(9) $$\begin{array}{cc} I_{u_{\nu }}\left(u_{\nu }\right) & =\left\{t\in \left\{1,2,\ldots ,n\right\}:\;u_{\nu ,t}=u_{\nu }\right\}. \end{array}$$

Within each subsequence of $u_{\nu }$ where $u_{\nu ,t}=u_{\nu }$, represented by $u_{\nu }\left(I_{u_{\nu }}\left(u_{\nu }\right)\right)$, symbols are drawn independently according to $Q_{\nu ,u_{\nu }}\left(x_{\nu }\right)$. For this mi-icd ensemble, codewords are generated according to

(10) $$\begin{array}{cc} Q_{\nu }^{\mathrm{mi}-\mathrm{icd}}\left(x_{\nu }|u_{\nu }\right) & =\prod_{u_{\nu }\in U_{\nu }}\prod_{t\in I_{u_{\nu }}\left(u_{\nu }\right)}Q_{\nu ,u_{\nu }}\left(x_{\nu ,t}\right) \end{array}$$

(11) $$\begin{array}{cc} \; & =\prod_{t=1}^{n}Q_{\nu ,u_{\nu ,t}}\left(x_{\nu ,t}\right). \end{array}$$

**Figure 1:**
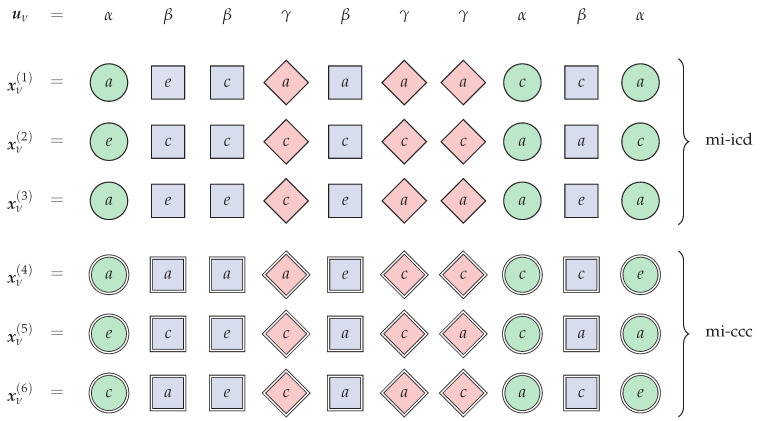
Example of codewords $x_{\nu }^{\left(1\right)}$, $x_{\nu }^{\left(2\right)}$ and $x_{\nu }^{\left(3\right)}$ in the mi-icd ensemble and $x_{\nu }^{\left(4\right)}$, $x_{\nu }^{\left(5\right)}$ and $x_{\nu }^{\left(6\right)}$ in the mi-ccc ensemble, for a given source sequence $u_{\nu }$.

Compared to the *mi-iid* ensemble, the *mi-icd* ensemble can lead to a larger transmissible region for the multiple-access channel with correlated sources [11,21]. An example of generation of three codewords $x_{\nu }^{\left(1\right)}$, $x_{\nu }^{\left(2\right)}$ and $x_{\nu }^{\left(3\right)}$ in the mi-icd ensemble is shown in Figure 1, for a given source sequence $u_{\nu }=\left(\alpha ,\beta ,\beta ,\gamma ,\beta ,\gamma ,\gamma ,\alpha ,\beta ,\alpha \right)$ with source alphabet U={α,β,γ}. To generate each codeword $x_{\nu }$ with alphabet X={a,c,e}, three subcodewords $x_{\nu }(I_{\alpha }\left(u_{\nu }\right)$, $x_{\nu }(I_{\beta }\left(u_{\nu }\right)$ and $x_{\nu }(I_{\gamma }\left(u_{\nu }\right)$ are pairwise-independently generated with i. i. d. distributions $Q_{\nu ,\alpha }=\left(1\backslash 3,1\backslash 3,1\backslash 3\right)$, $Q_{\nu ,\beta }=\left(1\backslash 2,1\backslash 4,1\backslash 4\right)$ and $Q_{\nu ,\gamma }=\left(1\backslash 3,2\backslash 3,0\right)$, respectively. Symbols generated according to $Q_{\nu ,\alpha }$, $Q_{\nu ,\beta }$ and $Q_{\nu ,\gamma }$ are respectively represented as green circles, blue boxes and red diamonds in the figure. In the example, $I_{\alpha }\left(u_{\nu }\right)=\left\{1,8,10\right\}$, $I_{\beta }\left(u_{\nu }\right)=\left\{2,3,5,9\right\}$ and $I_{\gamma }\left(u_{\nu }\right)=\left\{4,6,7\right\}$. For instance, the subcodeword $x_{\nu }^{\left(1\right)}(I_{\gamma }\left(u_{\nu }\right)$ has three symbols, each generated independently from $Q_{\nu ,\gamma }$, leading to the red-diamond symbols $x_{\nu }^{\left(1\right)}(I_{\gamma }\left(u_{\nu }\right)=\left(a,a,a\right)$.

Next, we have the message-dependent iid (*md-iid*) ensemble [6,8,19,25,28], where codewords for each user are generated with i. i. d. symbols according to different distributions $Q_{\nu }^{i_{\nu }}\left(x_{\nu }\right)$ that depend on the full source message through the class index $i_{\nu }$ of the class the source message belongs to. More precisely, for user ν=1,2 with source marginal distribution $P_{\nu }$, the $i_{\nu }$-th class $A_{\nu }^{i_{\nu }}$, where $i_{\nu }\in K_{\nu }=\left\{1,\ldots ,K_{\nu }\right\}$, is defined as the set of all source messages whose probability $P_{\nu }\left(u_{\nu }\right)$ is within a given interval, that is,

(12) $$\begin{array}{c} A_{\nu }^{i_{\nu }}=\left\{u_{\nu }\in U_{\nu }^{n}\;:\;\gamma_{\nu ,i_{\nu }}^{n}<P_{\nu }\left(u_{\nu }\right)\leq \gamma_{\nu ,i_{\nu }-1}^{n}\right\}, \end{array}$$

where the thresholds $\gamma_{\nu ,j}$ are $K_{\nu }+1$ non-negative numbers, ordered from higher to lower, such that $0=\gamma_{\nu ,K_{\nu }}\leq \gamma_{\nu ,K_{\nu }-1}\leq \ldots \leq \gamma_{\nu ,1}<\gamma_{\nu ,0}=1$, and $\min_{u_{\nu }}P_{\nu }\left(u_{\nu }\right)<\gamma_{\nu ,K_{\nu }-1}$ and $\gamma_{\nu ,1}\leq \max_{u_{\nu }}P_{\nu }\left(u_{\nu }\right)$. The *md-iid* random-coding distribution is given by

(13) $$\begin{array}{c} Q_{\nu }^{\mathrm{md}-\mathrm{iid}}\left(x_{\nu }|u_{\nu }\right)=\prod_{t=1}^{n}Q_{\nu }^{i_{\nu }\left(u_{\nu }\right)}\left(x_{\nu ,t}\right). \end{array}$$

The exponent of this *md-iid* ensemble can be larger than that of the *mi-iid* ensemble for joint source-channel coding [8,20,28].

In the message-dependent, independent conditional symbol distributions (*md-icd*) ensemble, messages in the class $i_{\nu }$ for user ν are encoded with codewords whose symbols are generated independently according to the conditional input distribution $Q_{\nu ,u_{\nu }}^{i_{\nu }}\left(x_{\nu }\right)$. The random-coding distribution of the *md-icd* ensemble is thus given by

(14) $$\begin{array}{c} Q_{\nu }^{\mathrm{md}-\mathrm{icd}}\left(x_{\nu }|u_{\nu }\right)=\prod_{u_{\nu }\in U_{\nu }}\prod_{t\in I_{u_{\nu }}\left(u_{\nu }\right)}Q_{\nu ,u_{\nu }}^{i_{\nu }\left(u_{\nu }\right)}\left(x_{\nu ,t}\right). \end{array}$$

In the message-independent, constant-composition (*mi-cc*) ensemble [29,30], codewords $x_{\nu }$ are drawn independently with an empirical distribution ${\hat{Q}}_{\nu }\left(x_{\nu }\right)$ close to a given $Q_{\nu }\left(x_{\nu }\right)$, independently of the source message $u_{\nu }$. For each user, codewords $x_{\nu }$ are randomly picked from $T_{\nu }^{n}\left(Q_{\nu }\right)$, the set of all sequences whose empirical distribution has a variational distance to $Q_{\nu }$ of at most 1\n, that is

(15) $$\begin{array}{c} T_{\nu }^{n}\left(Q_{\nu }\right)=\left\{x_{\nu }\in X_{\nu }^{n}:\max_{x_{\nu }}\;\left|{\hat{Q}}_{\nu }\left(x_{\nu }\right)-Q_{\nu }\left(x_{\nu }\right)\right|<\frac{1}{n}\right\}. \end{array}$$

For this *mi-cc* ensemble, the random-coding distribution is given by

(16) $$\begin{array}{c} Q_{\nu }^{\mathrm{mi}-\mathrm{cc}}\left(x_{\nu }|u_{\nu }\right)=\frac{1}{|T_{\nu }^{n}\left(Q_{\nu }\right)|}1\left\{x_{\nu }\in T_{\nu }^{n}\left(Q_{\nu }\right)\right\}. \end{array}$$

While the *mi-cc* and *mi-iid* ensembles lead to identical transmissibility conditions, the former may achieve strictly larger exponents for suboptimal input distributions already in single-user settings [29].

The message-independent, conditional constant-composition (*mi-ccc*) ensemble combines features of the *mi-icd* and *mi-cc* ensembles. For each subsequence $u_{\nu }\left(I_{u_{\nu }}\left(u_{\nu }\right)\right)$, the corresponding subcodewords $x_{\nu }\left(I_{u_{\nu }}\left(u_{\nu }\right)\right)$ are drawn independently from the set $T_{\nu }^{|I_{u_{\nu }}\left(u_{\nu }\right)|}\left(Q_{\nu ,u_{\nu }}\right)$ of subsequences with empirical distribution close to $Q_{\nu ,u_{\nu }}\left(x_{\nu }\right)$, namely

(17) $$\begin{array}{c} T_{\nu }^{|I_{u_{\nu }}\left(u_{\nu }\right)|}\left(Q_{\nu ,u_{\nu }}\right)=\left\{x_{\nu }\in X_{\nu }^{|I_{u_{\nu }}\left(u_{\nu }\right)|}:\max_{x_{\nu }\in x_{\nu }}\;\left|{\hat{Q}}_{\nu }\left(x_{\nu }\right)-Q_{\nu ,u_{\nu }}\left(x_{\nu }\right)\right|<\frac{1}{|I_{u_{\nu }}\left(u_{\nu }\right)|}\right\}. \end{array}$$

The random-coding distribution of the mi-ccc ensemble is given by

(18) $$\begin{array}{c} Q_{\nu }^{\mathrm{mi}-\mathrm{ccc}}\left(x_{\nu }|u_{\nu }\right)=\prod_{u_{\nu }\in U_{\nu }}\frac{1}{\left|T_{\nu }^{|I_{u_{\nu }}\left(u_{\nu }\right)|}\left(Q_{\nu ,u_{\nu }}\right)\right|}1\left\{x_{\nu }\left(I_{u_{\nu }}\left(u_{\nu }\right)\right)\in T_{\nu }^{|I_{u_{\nu }}\left(u_{\nu }\right)|}\left(Q_{\nu ,u_{\nu }}\right)\right\}. \end{array}$$

An example of the generation of three codewords $x_{\nu }^{\left(4\right)}$, $x_{\nu }^{\left(5\right)}$ and $x_{\nu }^{\left(6\right)}$ in the mi-ccc ensemble is also shown in Figure 1 as a comparison to the md-iid ensemble, for the same source sequence $u_{\nu }$, source alphabet U={α,β,γ} and input alphabet X={a,c,e}. Now, to generate each codeword $x_{\nu }$, three subcodewords $x_{\nu }\left(I_{\alpha }\left(u_{\nu }\right)\right)$, $x_{\nu }\left(I_{\beta }\left(u_{\nu }\right)\right)$ and $x_{\nu }\left(I_{\gamma }\left(u_{\nu }\right)\right)$ are pairwise-independently, uniformly drawn in the type classes with empirical distributions ${\hat{Q}}_{\nu ,\alpha }$, ${\hat{Q}}_{\nu ,\beta }$ and ${\hat{Q}}_{\nu ,\gamma }$ that are closest to $Q_{\nu ,\alpha }$, $Q_{\nu ,\beta }$ and $Q_{\nu ,\gamma }$, respectively. Since in the example $|I_{\alpha }\left(u_{\nu }\right)|=3$, $|I_{\beta }\left(u_{\nu }\right)|=4$ and $|I_{\gamma }\left(u_{\nu }\right)|=3$, it follows that ${\hat{Q}}_{\nu ,\alpha }=\left(1\backslash 3,1\backslash 3,1\backslash 3\right)$, ${\hat{Q}}_{\nu ,\beta }=\left(1\backslash 2,1\backslash 4,1\backslash 4\right)$ and ${\hat{Q}}_{\nu ,\gamma }=\left(1\backslash 3,2\backslash 3,0\right)$. Symbols generated according to ${\hat{Q}}_{\nu ,\alpha }$, ${\hat{Q}}_{\nu ,\beta }$ and ${\hat{Q}}_{\nu ,\gamma }$ are respectively represented as green doubled circles, blue doubled boxes and red doubled diamonds in the figure. For instance, all subcodewords $x_{\nu }^{\left(j\right)}(I_{\gamma }\left(u_{\nu })\right)$, for j=4,5,6, have three symbols jointly generated from the constant-composition type ${\hat{Q}}_{\nu ,\gamma }$, that is, exactly one *a* and two *c*s.

The message-dependent, constant-composition (*md-cc*) ensemble combines the features of having different distributions for different messages with constant-composition random coding. For messages in the class $i_{\nu }\in \left\{1,\ldots ,K_{\nu }\right\}$ for user ν, codewords are drawn from the set of sequences with empirical distribution close to $Q_{\nu }^{i_{\nu }}\left(x_{\nu }\right)$. For this ensemble, the random-coding distribution is given by

(19) $$\begin{array}{c} Q_{\nu }^{\mathrm{md}-\mathrm{cc}}\left(x_{\nu }|u_{\nu }\right)=\frac{1}{\left|T_{\nu }^{n}\left(Q_{\nu }^{i_{\nu }\left(u_{\nu }\right)}\right)\right|}1\{x_{\nu }\in T_{\nu }^{n}(Q_{\nu }^{i_{\nu }\left(u_{\nu }\right)})\}. \end{array}$$

Finally, the message-dependent, conditional constant-composition (*md-ccc*) ensemble combines several of the ensembles listed above. For a given message $u_{\nu }=\left(u_{\nu ,1},\ldots ,u_{\nu ,n}\right)$ in the $i_{\nu }$-th class, that is, $u_{\nu }\in A_{\nu }^{i_{\nu }}$, the subsequence of $u_{\nu }$ having the same symbol $u_{\nu }$, that is, $u_{\nu }\left(I_{u_{\nu }}\left(u_{\nu }\right)\right)$, is encoded with pairwise-independent codewords generated from the set of codewords with empirical distribution very close to $Q_{\nu ,u_{\nu }}^{i_{\nu }}\left(x_{\nu }\right)$. The random-coding distribution of the md-ccc ensemble is thus given by

(20) $$\begin{array}{c} Q_{\nu }^{\mathrm{md}-\mathrm{ccc}}\left(x_{\nu }|u_{\nu }\right)=\prod_{u_{\nu }\in U_{\nu }}\frac{1}{\left|T_{\nu }^{|I_{u_{\nu }}\left(u_{\nu }\right)|}\left(Q_{\nu ,u_{\nu }}^{i_{\nu }\left(u_{\nu }\right)}\right)\right|}1\left\{x_{\nu }\left(I_{u_{\nu }}\left(u_{\nu }\right)\right)\in T_{\nu }^{|I_{u_{\nu }}\left(u_{\nu }\right)|}\left(Q_{\nu ,u_{\nu }}^{i_{\nu }\left(u_{\nu }\right)}\right)\right\}. \end{array}$$

### 3.2. Generalized Multi-Class Cost-Constrained Ensemble

Motivated by the ensembles listed in the previous section, and inspired by refs. [1] (Ch. 7) and [26] (Sec. II), we study a generalized message-dependent multi-class cost-constrained random-coding ensemble with multiple auxiliary costs.

For each user, we partition the set of source messages into $K_{\nu }$ disjoint classes with thresholds on the message probabilities as in Equation (12). Let the source message be in the $i_{\nu }$-th class, that is, $i_{\nu }\left(u_{\nu }\right)=i_{\nu }$. Given the source message $u_{\nu }$ and the source symbol $u_{\nu }$, we consider the subsequence $u_{\nu }\left(I_{u_{\nu }}\left(u_{\nu }\right)\right)$, where $I_{u_{\nu }}\left(u_{\nu }\right)$ is defined in Equation (9), and we denote the corresponding source subsequence and subcodeword by $u_{\nu }\left(I_{u_{\nu }}\left(u_{\nu }\right)\right)$ and $x_{\nu }\left(I_{u_{\nu }}\left(u_{\nu }\right)\right)$ respectively. For each user ν, class index $i_{\nu }$, and source message symbol $u_{\nu }$, the subcodeword $x_{\nu }\left(I_{u_{\nu }}\left(u_{\nu }\right)\right)$ is drawn according to a symbolwise i. i. d. distribution $Q_{\nu ,u_{\nu }}^{i_{\nu }}\left(x_{\nu }\right)$ conditioned on a set of cost constraints being satisfied. We consider $L_{\nu }$ additive cost functions $a_{\nu ,u_{\nu }}^{i_{\nu },\ell_{\nu }}\left(x_{\nu }\right)$, $\ell_{\nu }\in L_{\nu }=\left\{1,\ldots ,L_{\nu }\right\}$. The total cost $a_{\nu ,u_{\nu }}^{i_{\nu },\ell_{\nu }}\left(x_{\nu }\left(I_{u_{\nu }}\left(u_{\nu }\right)\right)\right)$ of the subcodeword $x_{\nu }\left(I_{u_{\nu }}\left(u_{\nu }\right)\right)$ is given by the sum of the symbol costs $a_{\nu ,u_{\nu }}^{i_{\nu },\ell_{\nu }}$, namely

(21) $$\begin{array}{c} a_{\nu ,u_{\nu }}^{i_{\nu },\ell_{\nu }}\left(x_{\nu }\left(I_{u_{\nu }}\left(u_{\nu }\right)\right)\right)=\sum_{j\in I_{u_{\nu }}\left(u_{\nu }\right)}a_{\nu ,u_{\nu }}^{i_{\nu },\ell_{\nu }}\left(x_{\nu ,j}\right). \end{array}$$

We assume that the average cost $\phi_{\nu ,u_{\nu }}^{i_{\nu },\ell_{\nu }}$ under the conditional distribution $Q_{\nu ,u_{\nu }}^{i_{\nu }}$ is zero:

(22) $$\begin{array}{c} \phi_{\nu ,u_{\nu }}^{i_{\nu },\ell_{\nu }}=\sum_{x_{\nu }\in X_{\nu }}Q_{\nu ,u_{\nu }}^{i_{\nu }}\left(x_{\nu }\right)a_{\nu ,u_{\nu }}^{i_{\nu },\ell_{\nu }}\left(x_{\nu }\right)=0. \end{array}$$

Finally, fix some parameters $\delta_{\nu }>0$ and let $D_{\nu }^{i_{\nu }}$ be the set of codewords for which the average empirical cost of its constituent subcodewords $\frac{1}{|I_{u_{\nu }}\left(u_{\nu }\right)|}a_{\nu ,u_{\nu }}^{i_{\nu },\ell_{\nu }}\left(x_{\nu }\left(I_{u_{\nu }}\left(u_{\nu }\right)\right)\right)$ is close to the statistical mean $\phi_{\nu ,u_{\nu }}^{i_{\nu },\ell_{\nu }}=0$ for all cost functions and source symbols, i.e.,

(23) $$\begin{array}{c} D_{\nu ,u_{\nu }}^{i_{\nu }}≜\left\{x_{\nu }\in X_{\nu }^{n}:\left|\frac{1}{|I_{u_{\nu }}\left(u_{\nu }\right)|}a_{\nu ,u_{\nu }}^{i_{\nu },\ell_{\nu }}\left(x_{\nu }\left(I_{u_{\nu }}\left(u_{\nu }\right)\right)\right)\right|\leq \frac{\delta_{\nu }}{|I_{u_{\nu }}\left(u_{\nu }\right)|},\;u_{\nu }\in U_{\nu },\;\ell_{\nu }\in L_{\nu }\right\}. \end{array}$$

Codewords $x_{\nu }$ are the combination of subcodewords $x_{\nu }\left(I_{u_{\nu }}\left(u_{\nu }\right)\right)$ with respective positions in $I_{u_{\nu }}\left(u_{\nu }\right)$. For this multi-class cost-constrained ensemble, the random-coding distribution is thus given by

(24) $$\begin{array}{cc} Q_{\nu }^{\mathrm{cost}}\left(x_{\nu }|u_{\nu }\right)\; & =\frac{1}{\Xi_{\nu }}\prod_{u_{\nu }\in U_{\nu }}\prod_{t\in I_{u_{\nu }}\left(u_{\nu }\right)}Q_{\nu ,u_{\nu }}^{i_{\nu }}\left(x_{\nu ,t}\right)1\left\{x_{\nu }\in D_{\nu ,u_{\nu }}^{i_{\nu }}\right\} \end{array}$$

(25) $$\begin{array}{cc} \; & =\frac{1}{\Xi_{\nu }}\prod_{t=1}^{n}Q_{\nu ,u_{\nu ,t}}^{i_{\nu }}\left(x_{\nu ,t}\right)1\left\{x_{\nu }\in D_{\nu ,u_{\nu }}^{i_{\nu }}\right\}, \end{array}$$

where $\Xi_{\nu }$ is a normalizing constant and the class index is determined by the source message, $i_{\nu }=i_{\nu }\left(u_{\nu }\right)$.

The multi-class cost-constrained ensemble subsumes all the ensembles described in Section 3.1. First of all, the *iid* and *icd* ensembles are recovered by setting $L_{\nu }=0$ and choosing the appropriate number of classes $K_{\nu }$ and random-coding distributions $Q_{\nu }$, $Q_{\nu ,u_{\nu }}$, $Q_{\nu }^{i_{\nu }}$ and $Q_{\nu ,u_{\nu }}^{i_{\nu }}$. For all these cases, the set $D_{\nu ,u_{\nu }}^{i_{\nu }}$ includes all generated codewords and the normalizing constant is $\Xi_{\nu }=1$.

To recover the constant-composition ensembles, for which constraints force the subcodewords to belong to some set $T_{\nu }^{n}\left(Q_{\nu }\right)$ or $T_{\nu }^{|I_{u_{\nu }}\left(u_{\nu }\right)|}\left(Q_{\nu ,u_{\nu }}^{i_{\nu }}\right)$, for each of the $K_{\nu }$ classes for user ν we set $\delta_{\nu }<1$, $L_{\nu }=\left|X_{\nu }\right|$ and bijectively map the channel input symbols to cost function indices $\ell_{\nu }\left(x_{\nu }\right)$ so that

(26) $$\begin{array}{c} a_{\nu ,u_{\nu }}^{i_{\nu },\ell_{\nu }}\left(x_{\nu }\right)=1\left\{x_{\nu }=\ell_{\nu }\right\}-Q_{\nu ,u_{\nu }}^{i_{\nu }}\left(\ell_{\nu }\right). \end{array}$$

In case the ensemble does not depend on either $i_{\nu }$ or $u_{\nu }$, these symbols are dropped from Equation (26). For example, for the md-cc ensemble, we have $a_{\nu ,\ell_{\nu }}^{i_{\nu }}\left(\ell_{\nu }\right)=1\left\{x_{\nu }=\ell_{\nu }\right\}-Q_{\nu }^{i_{\nu }}\left(x_{\nu }\right)$. In addition, the codeword set $D_{\nu ,u_{\nu }}^{i_{\nu }}$ in Equation (23) is simplified as

(27) $$\begin{array}{c} D_{\nu ,u_{\nu }}^{i_{\nu }}=\left\{x_{\nu }\in X_{\nu }^{n}:\left|\frac{1}{n}\sum_{t=1}^{n}1\left\{x_{\nu ,t}=x\right\}-Q_{\nu }^{i_{\nu }}\left(x\right)\right|\leq \frac{1}{n},\;x\in X_{\nu }\right\}, \end{array}$$

which is the same as $T^{n}\left(Q_{\nu }^{i_{\nu }}\right)$ given a version of Equation (15) where $Q_{\nu }$ may depend on $i_{\nu }$.

Again, choosing the right number of classes $K_{\nu }$ and random-coding distributions $Q_{\nu }$, $Q_{\nu ,u_{\nu }}$, $Q_{\nu }^{i_{\nu }}$, and $Q_{\nu ,u_{\nu }}^{i_{\nu }}$ recovers the various constant-composition ensembles. By construction, the set $D_{\nu ,u_{\nu }}^{i_{\nu }}$ includes only the (sub)codewords with empirical distribution close to respectively $Q_{\nu }$, $Q_{\nu ,u_{\nu }}$, $Q_{\nu }^{i_{\nu }}$, and $Q_{\nu ,u_{\nu }}^{i_{\nu }}$, and the normalizing constant $\Xi_{\nu }$ is the probability of the corresponding type set (or product thereof). As an example, for the md-ccc ensemble, choosing the cost functions in Equation (26) as follows

(28) $$\begin{array}{c} a_{\nu ,u_{\nu }}^{i_{\nu },\ell_{\nu }}\left(x_{\nu }\left(I_{u_{\nu }}\left(u_{\nu }\right)\right)\right)=\sum_{j\in I_{u_{\nu }}\left(u_{\nu }\right)}1\left\{x_{\nu ,j}=\ell_{\nu }\right\}-Q_{\nu ,u_{\nu }}^{i_{\nu }}\left(\ell_{\nu }\right) \end{array}$$

yields the following cost-constraint set, which is equivalent to Equation (17),

(29) $$\begin{array}{c} D_{\nu ,u_{\nu }}^{i_{\nu }}=\left\{x_{\nu }\in X_{\nu }^{n}:|\frac{\sum_{j\in I_{u}\left(u_{\nu }\right)}1\left\{x_{\nu ,j}=x\right\}}{\left|I_{u}\left(u_{\nu }\right)\right|}-Q_{\nu ,u_{\nu }}^{i_{\nu }}\left(x\right)|\leq \frac{1}{\left|I_{u}\left(u_{\nu }\right)\right|},\;u\in U_{\nu },\;x\in X_{\nu }\right\}. \end{array}$$

### 3.3. Exponent for the Generalized Multi-Class Cost-Constrained Ensemble

**Theorem** **1.** *For the transmission of N correlated memorlyess sources with joint distribution $P_{N}$, where N={1,2,…,N}, over a channel with input $x_{N}$ over a memoryless channel with transition probabilitiy $W(y|x_{N})$, consider a random-coding multi-class cost-constrained ensemble where source messages for each user ν∈N are allocated, depending on their probabilities, into $K_{\nu }$ classes with thresholds $\left\{\gamma_{\nu ,0},\gamma_{\nu ,1},\ldots ,\gamma_{\nu ,K_{\nu }}\right\}$, as in Equation (12), and encoded onto codewords randomly generated with a distribution $Q_{\nu }^{i_{\nu }}\left(x_{\nu }|u_{\nu }\right)$ that depends on the source message according to Equation (24) through symbol distributions $Q_{\nu ,u_{\nu }}^{i_{\nu }}$ that possibly depend on the source-message class index $i_{\nu }$ and source symbol $u_{\nu }$ and $L_{\nu }$ cost functions $a_{\nu ,u_{\nu }}^{i_{\nu },\ell_{\nu }}$, $\ell_{\nu }\in \left\{1,2,\ldots ,L_{\nu }\right\}$. This random-coding ensemble attains the following exponent $E^{\cos t}$*

(30) $$\begin{array}{cc} E^{\mathrm{cost}} & =\min_{\begin{array}{c} \tau \in 2^{N}\backslash \emptyset ,\;i_{N}\in K_{N} \end{array}}\;\max_{0\leq \rho \leq 1}\;\max_{\begin{array}{c} \lambda_{N}^{L,U}\geq 0,\;r_{Nu_{N}}^{\ell_{N}}\in R \end{array}}E_{\tau }^{i_{N}}\left(\rho ,\lambda_{N}^{L,U},r_{Nu_{N}}^{\ell_{N}}\right), \end{array}$$

*where the Gallager function $E_{\tau }^{i_{N}}\left(\rho ,\lambda_{N}^{L,U},r_{Nu_{N}}^{\ell_{N}}\right)$ is given by*

(31) $$\begin{array}{cc} \; & \;E_{\tau }^{i_{N}}\left(\rho ,\lambda_{N}^{L,U},r_{Nu_{N}}^{\ell_{N}}\right)=\\ \; & \;-\log\sum_{u_{\tau^{c}},x_{\tau^{c}},y}{\left(\sum_{u_{\tau },x_{\tau }}P_{N}{\left(u_{N}\right)}^{\frac{1}{1+\rho }}\Lambda_{N}^{i_{N}}\left(u_{N}\right)Q_{\tau ,u_{\tau }}^{i_{\tau }}\left(x_{\tau }\right)R_{N,u_{N}}^{i_{N}}\left(x_{N}\right){\left(Q_{\tau^{c},u_{\tau^{c}}}^{i_{\tau^{c}}}\left(x_{\tau^{c}}\right)W\left(y|x_{N}\right)\right)}^{\frac{1}{1+\rho }}\right)}^{\;\;1+\rho }, \end{array}$$

*and the functions $\Lambda_{\sigma }^{i_{\sigma }}\left(u_{\sigma }\right)$ and $R_{\sigma ,u_{\sigma }}^{i_{\sigma }}\left(x_{\sigma }\right)$ are respectively given by*

(32) $$\begin{array}{c} \Lambda_{\sigma }^{i_{\sigma }}\left(u_{\sigma }\right)=\prod_{\nu \in \sigma }{\left(\frac{P_{\nu }\left(u_{\nu }\right)}{\gamma_{\nu ,i_{\nu }}}\right)}^{\lambda_{\nu }^{L}}{\left(\frac{\gamma_{\nu ,i_{\nu }-1}}{P_{\nu }\left(u_{\nu }\right)}\right)}^{\lambda_{\nu }^{U}}, \end{array}$$

(33) $$\begin{array}{c} R_{\sigma ,u_{\sigma }}^{i_{\sigma }}\left(x_{\sigma }\right)=\prod_{\nu \in \sigma }\prod_{\ell_{\nu }\in L_{\nu }}e^{r_{\nu u_{\nu }}^{\ell_{\nu }}\;a_{\nu ,u_{\nu }}^{i_{\nu },\ell_{\nu }}\left(x_{\nu }\right)}, \end{array}$$

*and implicitly depende on the set of optimization parameters $\left(\lambda_{N}^{L,U},r_{Nu_{N}}^{\ell_{N}}\right)$.*

**Proof.**  This result is proved in Appendix A. □

The random-coding exponent in Equation (30) depends on the partitioning of the source-message set into classes, the channel input distributions, and the codeword cost-constraint functions. The best possible generalized cost-constraint exponent is obtained by optimizing over the multi-class partitioning, the cost constraints and the input distributions. We briefly discuss the optimization w. r. t. the thresholds of the source messages partitioning in Appendix B. In the next section, we provide some numerical examples where we compute the optimal exponents for either independent or correlated sources, and find that the optimal number of classes is two. In ref. [31] (Sec. 3.2.1.1), we provide some indications of why this optimality of only two classes is harder to establish in multi-user scenarios, compared to the single-user case. In the next section, we use Equations (31) and (30) to respectively obtain the source and channel Gallager functions of the various ensembles in Section 3.1 and rank their achievable exponents and transmissibility regions.

## 4. Discussion

### 4.1. Gallager Functions for Correlated Sources

In this section, we evaluate the generalized Gallager function $E_{\tau }^{i_{N}}\left(\rho ,\lambda_{N}^{L,U},r_{Nu_{N}}^{\ell_{N}}\right)$ of the multi-class cost-constrained ensemble in Equation (31) for the various ensembles described in Section 3.1. In the cases where it is possible, we relate this Gallager function to the well-known [1] correlated-source and channel Gallager functions, respectively given by:

(34) $$\begin{array}{c} E_{s,\sigma }\left(\rho ,P_{N}\right)=\log\sum_{u_{\sigma^{c}}}{\left(\sum_{u_{\sigma }}P_{N}{\left(u_{N}\right)}^{\frac{1}{1+\rho }}\right)}^{1+\rho }, \end{array}$$

(35) $$\begin{array}{c} E_{0}\left(\rho ,Q,W\right)=-\log\sum_{y}{\left(\sum_{x}Q\left(x\right)W{\left(y|x\right)}^{\frac{1}{1+\rho }}\right)}^{1+\rho }, \end{array}$$

where $\sigma \in 2^{N}$. Using that $\left[u_{\sigma },u_{\sigma^{c}}\right]=u_{N}$, the standard Gallager source function is given by $E_{s}\left(\rho ,P_{N}\right)=E_{s,N}\left(\rho ,P_{N}\right)$, with N={1,…,N} the set of user indices.

For the simple mi-iid ensemble, with only one source class and no cost constraints, $K_{\nu }=1$ and $L_{\nu }=0$ for all ν∈N, and $\Lambda_{\sigma }^{i_{\sigma }}\left(u_{\sigma }\right)=R_{\sigma ,u_{\sigma }}^{i_{\sigma }}\left(x_{\sigma }\right)=1$ for all $\sigma \in 2^{N}$. With no statistical dependency between messages and codewords, $Q_{\nu ,u_{\nu }}\left(x_{\nu }\right)=Q_{\nu }\left(x_{\nu }\right)$. Setting $i_{N}=1$ and $\lambda_{N}^{L,U}=r_{Nu_{N}}^{\ell_{N}}=0$ in Equation (31) gives the Gallager function $E_{\tau }^{\mathrm{mi}-\mathrm{iid}}\left(\rho ,P_{N},Q_{N},W\right)$,

(36) $$\begin{array}{cc} E_{\tau }^{\mathrm{mi}-\mathrm{iid}} & \left(\rho ,P_{N},Q_{N},W\right)\\ \; & =-\log\sum_{u_{\tau^{c}},x_{\tau^{c}},y}{\left(\sum_{u_{\tau },x_{\tau }}P_{N}{\left(u_{N}\right)}^{\frac{1}{1+\rho }}Q_{\tau }\left(x_{\tau }\right){\left(Q_{\tau^{c}}\left(x_{\tau^{c}}\right)W\left(y|x_{N}\right)\right)}^{\frac{1}{1+\rho }}\right)}^{1+\rho }. \end{array}$$

Isolating the summations over $u_{\tau^{c}}$ and $u_{\tau }$, we can split the Gallager function as

(37) $$\begin{array}{cc} E_{\tau }^{\mathrm{mi}-\mathrm{iid}}\left(\rho ,P_{N},Q_{N},W\right) & =E_{0}\left(\rho ,Q_{\tau },Q_{\tau^{c}}W\right)-E_{s,\tau }\left(\rho ,P_{N}\right), \end{array}$$

where $Q_{\tau^{c}}W$ is a shorthand for $Q_{\tau^{c}}\left(x_{\tau^{c}}\right)W\left(y|x_{N}\right)$, the transition probability of a channel with input $x_{\tau }$ and output $\left(x_{\tau^{c}},y\right)$.

For the *mi-icd* ensemble, we have a similar set-up as for the *mi-iid* ensemble, where $Q_{\nu ,u_{\nu }}\left(x_{\nu }\right)$ now may depend on $u_{\nu }$. In this case, the Gallager function $E_{\tau }^{\mathrm{mi}-\mathrm{icd}}\left(\cdot \right)$ is given by Equation (36) with $Q_{\sigma }\left(x_{\sigma }\right)$ replaced by $Q_{\sigma ,u_{\sigma }}\left(x_{\sigma }\right)$, for $\sigma \in \{\tau ,\tau^{c}\}$:

(38) $$\begin{array}{cc} E_{\tau }^{\mathrm{mi}-\mathrm{icd}} & \left(\rho ,P_{N},Q_{N,U},W\right)\\ \; & =-\log\sum_{u_{\tau^{c}},x_{\tau^{c}},y}{\left(\sum_{u_{\tau },x_{\tau }}P_{N}{\left(u_{N}\right)}^{\frac{1}{1+\rho }}Q_{\tau ,u_{\tau }}\left(x_{\tau }\right){\left(Q_{\tau^{c},u_{\tau^{c}}}\left(x_{\tau^{c}}\right)W\left(y|x_{N}\right)\right)}^{\frac{1}{1+\rho }}\right)}^{1+\rho }. \end{array}$$

As the summations over $u_{\tau^{c}}$ and $u_{\tau }$ are not independent from the rest, the Gallager function does not split into source and channel functions unless the sources are independent, in which case one can find an *mi-iid* ensemble with a tilted unconditional input distribution and identical exponent. To this end, and for a given conditional input distribution $Q_{\nu ,u_{\nu }}\left(x_{\nu }\right)$, let us define a tilted distribution $Q_{\nu }^{\rho }\left(x_{\nu }\right)$ as

(39) $$\begin{array}{c} Q_{\nu }^{\rho }\left(x_{\nu }\right)=\sum_{u_{\nu }}\frac{P_{\nu }{\left(u_{\nu }\right)}^{\frac{1}{1+\rho }}}{\sum_{{\bar{u}}_{\nu }}P_{\nu }{\left({\bar{u}}_{\nu }\right)}^{\frac{1}{1+\rho }}}Q_{\nu ,u_{\nu }}\left(x_{\nu }\right). \end{array}$$

From this equation, we have the following equality:

(40) $$\begin{array}{c} Q_{\nu }^{\rho }\left(x_{\nu }\right)\sum_{{\bar{u}}_{\nu }}P_{\nu }{\left({\bar{u}}_{\nu }\right)}^{\frac{1}{1+\rho }}=\sum_{u_{\nu }}P_{\nu }{\left(u_{\nu }\right)}^{\frac{1}{1+\rho }}Q_{\nu ,u_{\nu }}\left(x_{\nu }\right). \end{array}$$

Substituting this identity together with $P_{N}\left(u_{N}\right)=P_{\tau }\left(u_{\tau }\right)P_{\tau^{c}}\left(u_{\tau^{c}}\right)$ in Equation (38) and rearranging the result, we obtain the following Gallager function for independent sources:

(41) $$\begin{array}{cc} E_{\tau }^{\mathrm{mi}-\mathrm{icd}}\left(\rho ,P_{N},Q_{N,U},W\right) & =E_{0}\left(\rho ,Q_{\tau }^{\rho },WQ_{\tau^{c}}\right)-E_{s}\left(\rho ,P_{\tau }\right) \end{array}$$

(42) $$\begin{array}{cc} \; & =E_{\tau }^{\mathrm{mi}-\mathrm{iid}}\left(\rho ,P_{N},\left[Q_{\tau }^{\rho },Q_{\tau^{c}}\right],W\right). \end{array}$$

For the *md-iid* and *md-icd* ensembles, there are $K_{\nu }$ source classes per user and no cost constraints, i.e., $L_{\nu }=0$ and $R_{\sigma ,u_{\sigma }}^{i_{\sigma }}\left(x_{\sigma }\right)=1$ for ν∈N and $\sigma \in 2^{N}$. Settting $r_{Nu_{N}}^{\ell_{N}}=0$ in Equation (31) gives the Gallager function $E_{\tau ,i_{N}}^{\mathrm{md}-\mathrm{icd}}\left(\cdot \right)$ for generic $i_{N}$ [31] (Eq. (4.36)),

(43) $$\begin{array}{cc} E & {}_{\tau ,i_{N}}^{\mathrm{md}-\mathrm{icd}}\left(\rho ,P_{N},Q_{N,U}^{i_{N}},W\right)\\ \; & =-\log\sum_{u_{\tau^{c}},x_{\tau^{c}},y}{\left(\sum_{u_{\tau },x_{\tau }}P_{N}{\left(u_{N}\right)}^{\frac{1}{1+\rho }}\Lambda_{N}^{i_{N}}\left(u_{{}_{N}}\right)Q_{\tau ,u_{\tau }}^{i_{\tau }}\left(x_{\tau }\right){\left(Q_{\tau^{c},u_{\tau^{c}}}^{i_{\tau c}}\left(x_{\tau^{c}}\right)W\left(y|x_{N}\right)\right)}^{\frac{1}{1+\rho }}\right)}^{1+\rho }. \end{array}$$

The Gallager function $E_{\tau ,i_{N}}^{\mathrm{md}-\mathrm{iid}}\left(\cdot \right)$ for the *md-iid* ensemble is obtained by setting $Q_{\sigma ,u_{\sigma }}\left(x_{\sigma }\right)=Q_{\sigma }\left(x_{\sigma }\right)$, independent of $u_{\nu }$, for $\sigma \in \{\tau ,\tau^{c}\}$ in Equation (43). As the summations over $u_{\tau^{c}}$ and $u_{\tau }$ are now independent from the rest, the Gallager function splits as

(44) $$\begin{array}{cc} \; & E_{\tau ,i_{N}}^{\mathrm{md}-\mathrm{iid}}\left(\rho ,P_{N},Q_{N}^{i_{N}},W\right)=E_{0}\left(\rho ,Q_{\tau }^{i_{\tau }},Q_{\tau^{c}}^{i_{\tau^{c}}}W\right)-E_{s,\tau }^{i_{N}}\left(\rho ,P_{N}\right), \end{array}$$

where we defined $E_{s,\tau }^{i_{N}}\left(\rho ,P_{N}\right)$, a modified Gallager $E_{s}$-function, as

(45) $$\begin{array}{c} E_{s,\tau }^{i_{N}}\left(\rho ,P_{N}\right)=\log\sum_{u_{\tau^{c}}}{\left(\sum_{u_{\tau }}P_{N}{\left(u_{N}\right)}^{\frac{1}{1+\rho }}\Lambda_{N}^{i_{N}}\left(u_{N}\right)\right)}^{1+\rho }. \end{array}$$

The maximization w.r.t. $\lambda_{N}^{L,U}$ in Equation (30) only affects the second term in the r. h. s. of Equation (44), since the function $\Lambda_{N}^{i_{N}}$ only appears in the source part of the exponent. In Appendix C, we discuss the properties of Equation (45) after the maximization w.r.t. $\lambda_{N}^{L,U}$ as a function of ρ, and establish some connections to the Gallager source function (34) and to the source functions for the single-user md-iid ensemble in ref. [8].

The Gallager functions for the constant-composition ensembles differ from the ones considered so far in the presence of $L_{\nu }=\left|X_{\nu }\right|$ cost functions $a_{\nu ,u_{\nu }}^{i_{\nu },\ell_{\nu }}\left(x_{\nu }\right)$, given in Equation (26), for each input distribution $Q_{\nu ,u_{\nu }}^{i_{\nu }}\left(x_{\nu }\right)$. These cost functions appear in the Gallager functions through the factors $R_{\sigma ,u_{\sigma }}^{i_{\sigma }}\left(x_{\sigma }\right)$, for $\sigma \in \{\tau ,\tau^{c}\}$ that multiply each appearance of $Q_{\sigma ,u_{\sigma }}^{i_{\sigma }}\left(x_{\sigma }\right)$ in the function, and through their associated optimization parameters $r_{Nu_{N}}^{\ell_{N}}$. The expressions of the Gallager functions for these constant-composition ensembles can be easily inferred from this obversation, so we focus on the factor $R_{\sigma ,u_{\sigma }}^{i_{\sigma }}\left(x_{\sigma }\right)$ itself.

For the *mi-cc* and *md-cc* ensembles, the cost functions $a_{\nu ,u_{\nu }}^{i_{\nu },\ell_{\nu }}\left(x_{\nu }\right)$, factor $R_{\sigma ,u_{\sigma }}^{i_{\sigma }}\left(x_{\sigma }\right)$, and associated optimization parameter $r_{\nu u_{\nu }}^{\ell_{\nu }}$ are independent of $u_{\nu }$, we thus write $a_{\nu }^{i_{\nu },\ell_{\nu }}\left(x_{\nu }\right)$, $R_{\sigma }^{i_{\sigma }}\left(x_{\sigma }\right)$, and $r_{\nu }^{\ell_{\nu }}$. The expressions in Equations (26) and (33) for $L_{\nu }=X_{\nu }$ give

(46) $$\begin{array}{c} R_{\nu }^{i_{\nu }}\left(x_{\nu }\right)=e^{\sum_{\ell_{\nu }\in X_{\nu }}r_{\nu }^{\ell_{\nu }}\left(1\left\{x_{\nu }=\ell_{\nu }\right\}-Q_{\nu }^{i_{\nu }}\left(\ell_{\nu }\right)\right)}. \end{array}$$

The exponent in Equation (46) can be evaluated as

(47) $$\begin{array}{cc} \sum_{\ell_{\nu }\in X_{\nu }}r_{\nu }^{\ell_{\nu }}\left(1\left\{x_{\nu }=\ell_{\nu }\right\}-Q_{\nu }^{i_{\nu }}\left(\ell_{\nu }\right)\right) & =r_{\nu }^{x_{\nu }}-\sum_{\ell_{\nu }\in X_{\nu }}r_{\nu }^{\ell_{\nu }}Q_{\nu }^{i_{\nu }}\left(\ell_{\nu }\right) \end{array}$$

(48) $$\begin{array}{cc} \; & =\alpha_{\tau ,\nu }^{i_{\nu }}\left(x_{\nu }\right), \end{array}$$

where we have defined a function $\alpha_{\tau ,\nu }^{i_{\nu }}\left(x_{\nu }\right)$ that depends on τ and $i_{\nu }$ through the optimization parameters $r_{\nu }^{\ell_{\nu }}$. We can be easily verify that $\alpha_{\tau ,\nu }^{i_{\nu }}$ has zero mean, in other words, $\sum_{x_{\nu }}\alpha_{\tau ,\nu }^{i_{\nu }}\left(x_{\nu }\right)Q_{\nu }^{i_{\nu }}\left(x_{\nu }\right)=0$. At this point, the parameters $r_{\nu }^{\ell_{\nu }}$ may be replaced by the equivalent real-valued functions $\alpha_{\tau ,\nu }^{i_{\nu }}\left(x_{\nu }\right)$. We obtain the *mi-cc* Gallager function $E_{\tau }^{\mathrm{mi}-\mathrm{cc}}\left(\cdot \right)$ by setting $i_{N}=1$ and $\lambda_{N}^{L,U}=0$ in Equation (31),

$$\begin{array}{l} E{}_{\tau }^{\mathrm{mi}-\mathrm{cc}}\left(\rho ,\alpha_{\tau ,N},P_{N},Q_{N},W\right) \end{array}$$

(49) $$\begin{array}{cc} \; & =-\log\sum_{u_{\tau^{c}},x_{\tau^{c}},y}{\left(\sum_{u_{\tau },x_{\tau }}P_{N}{\left(u_{N}\right)}^{\frac{1}{1+\rho }}Q_{\tau }\left(x_{\tau }\right)e^{\alpha_{\tau ,N}\left(x_{N}\right)}{\left(Q_{\tau^{c}}\left(x_{\tau^{c}}\right)W\left(y|x_{N}\right)\right)}^{\frac{1}{1+\rho }}\right)}^{1+\rho } \end{array}$$

(50) $$\begin{array}{cc} \; & =-\log\sum_{x_{\tau^{c}},y}{\left(\sum_{x_{\tau }}Q_{\tau }\left(x_{\tau }\right)e^{\alpha_{\tau ,N}\left(x_{N}\right)}{\left(Q_{\tau^{c}}\left(x_{\tau^{c}}\right)W\left(y|x_{N}\right)\right)}^{\frac{1}{1+\rho }}\right)}^{1+\rho }-E_{s,\tau }\left(\rho ,P_{N}\right), \end{array}$$

where we split the Gallager function into channel and source terms in analogy to Equation (37).

In ref. [31] (Eq. (4.49)), the *md-cc* ensemble was studied for N=2 users in both the primal and dual domains. The md-cc Gallager function $E_{\tau }^{\mathrm{md}-\mathrm{cc}}\left(\cdot \right)$ for *N* users is obtained by combining the derivation of Equation (50) with that of Equation (44) to yield

(51) $$\begin{array}{cc} E_{\tau }^{\mathrm{md}-\mathrm{cc}}\left(\rho ,\alpha_{\tau ,N}^{i_{N}},P_{N},Q_{N}^{i_{N}},W\right)=-\log & \sum_{x_{\tau^{c}},y}{\left(\sum_{x_{\tau }}Q_{\tau }\left(x_{\tau }\right)e^{\alpha_{\tau ,N}^{i_{N}}\left(x_{N}\right)}{\left(Q_{\tau^{c}}\left(x_{\tau^{c}}\right)W\left(y|x_{N}\right)\right)}^{\frac{1}{1+\rho }}\right)}^{1+\rho }\\ & -\log\sum_{u_{\tau^{c}}}{\left(\sum_{u_{\tau }}P_{N}{\left(u_{N}\right)}^{\frac{1}{1+\rho }}\Lambda_{N}^{i_{N}}\left(u_{{}_{N}}\right)\right)}^{1+\rho }. \end{array}$$

As in previous cases, the exponent is obtained after maximization over $\alpha_{\tau ,N}^{i_{N}}$.

Concluding our list, the cost functions $a_{\nu ,u_{\nu }}^{i_{\nu },\ell_{\nu }}\left(x_{\nu }\right)$, factors $R_{\sigma ,u_{\sigma }}^{i_{\sigma }}\left(x_{\sigma }\right)$, and parameters $r_{\nu u_{\nu }}^{\ell_{\nu }}$ for the *mi-ccc* and *md-ccc* ensembles do depend on $u_{\nu }$. In analogy to Equation (48), we define a zero-mean function $\beta_{\tau ,\nu ,u_{\nu }}^{i_{\nu }}\left(x_{\nu }\right)$ as

(52) $$\begin{array}{c} \beta_{\tau ,\nu ,u_{\nu }}^{i_{\nu }}\left(x_{\nu }\right)=r_{\nu u_{\nu }}^{x_{\nu }}-\sum_{\ell_{\nu }\in X_{\nu }}r_{\nu u_{\nu }}^{\ell_{\nu }}Q_{\nu ,u_{\nu }}^{i_{\nu }}\left(\ell_{\nu }\right), \end{array}$$

and similarly for $\beta_{\tau ,\nu ,u_{\nu }}\left(x_{\nu }\right)$ for the *mi-ccc* ensemble. The Gallager function for the *mi-ccc* ensemble $E_{\tau }^{\mathrm{mi}-\mathrm{ccc}}\left(\cdot \right)$ is obtained by combining the derivations of Equation (50) and of Equation (38),

(53) $$\begin{array}{c} E_{\tau }^{\mathrm{mi}-\mathrm{ccc}}\left(\rho ,\beta_{\tau ,N,u_{N}},P_{N},Q_{N,U},W\right)\\ =-\log\sum_{u_{\tau^{c}},x_{\tau^{c}},y}{\left(\sum_{u_{\tau },x_{\tau }}P_{N}{\left(u_{N}\right)}^{\frac{1}{1+\rho }}Q_{\tau ,u_{\tau }}\left(x_{\tau }\right)e^{\beta_{\tau ,N,u_{N}}\left(x_{N}\right)}{\left(Q_{\tau^{c},u_{\tau^{c}}}\left(x_{\tau^{c}}\right)W\left(y|x_{N}\right)\right)}^{\frac{1}{1+\rho }}\right)}^{1+\rho }. \end{array}$$

Similarly, for the *md-ccc* ensemble, and in agreeement with the 2-user case studied in ref. [31] (Eq. (4.45)), combining the derivations of Equations (50) and (43), yields

(54) $$\begin{array}{cc} \; & \;E_{\tau ,i_{N}}^{\mathrm{md}-\mathrm{ccc}}\left(\rho ,\beta_{\tau ,N,u_{N}}^{i_{N}},P_{N},Q_{N,U}^{i_{N}},W\right)\\ \; & \;=-\log\sum_{u_{\tau^{c}},x_{\tau^{c}},y}{\left(\sum_{u_{\tau },x_{\tau }}P_{N}{\left(u_{N}\right)}^{\frac{1}{1+\rho }}\Lambda_{N}^{i_{N}}\left(u_{{}_{N}}\right)Q_{\tau ,u_{\tau }}^{i_{\tau }}\left(x_{\tau }\right)e^{\beta_{\tau ,N,u_{N}}^{i_{N}}\left(x_{N}\right)}{\left(Q_{\tau^{c},u_{\tau^{c}}}^{i_{\tau c}}\left(x_{\tau^{c}}\right)W\left(y|x_{N}\right)\right)}^{\frac{1}{1+\rho }}\right)}^{1+\rho }. \end{array}$$

### 4.2. Transmissibility

We may obtain the transmissibility conditions from the achievable exponents derived in Section 4.1, following the random-coding method described in ref. [1] (Th. 5.6.4). The analysis extends the transmissibility condition for joint source-channel coding in ref. [1] (Prob. 5.16), to account for statistical dependency of the codeword on the source message in the multiuser set-up. As mentioned above, the source $U_{N}$ is transmissible over the channel *W* if there exists a sequence of codes with vanishing error probability, or equivalently, with strictly positive achievable error exponent $E^{cost}$ in Equation (30). As an example, we present the derivation for the mi-icd ensemble where the class and cost functions in Equations (32) and (33) are inactive, namely $\Lambda_{\sigma }^{i_{\sigma }}\left(u_{\sigma }\right)=R_{\sigma ,u_{\sigma }}^{i_{\sigma }}\left(x_{\sigma }\right)=1$ for all $\sigma \in 2^{N}$, and leave the general case of $K_{\nu }>1$ classes and cost-constrained codewords as an open problem.

For the *mi-icd* case, and similarly to Gallager’s $E_{0}$-function [1] (Th. 5.6.3), the Gallager function $E_{\tau }^{\mathrm{mi}-\mathrm{icd}}\left(\cdot \right)$ in Equation (38) is concave (∩) with respect to ρ and satisfies $E_{\tau }^{\mathrm{mi}-\mathrm{icd}}\left(\rho =0,\cdot \right)=0$. For every $\tau \subset 2^{N}\backslash \emptyset$, let ${\hat{\rho }}_{\tau }$ be the optimizer given by

(55) $${\hat{\rho }}_{\tau }=\underset{0\leq \rho \leq 1}{arg max}E_{\tau }^{\mathrm{mi}-\mathrm{icd}}\left(\rho ,P_{N},Q_{N,U},W\right).$$

Therefore, the achievable exponent is strictly positive, namely $E_{\tau }^{\mathrm{mi}-\mathrm{icd}}\left({\hat{\rho }}_{\tau },\cdot \right)>0$, as far as the slope of the $E_{\tau }^{\mathrm{mi}-\mathrm{icd}}\left(\rho ,\cdot \right)$ function is strictly positive at ρ=0, that is

(56) $$\frac{\partial }{\partial \rho }{\left.E_{\tau }^{\mathrm{mi}-\mathrm{icd}}\left(\rho ,P_{N},Q_{N,U},W\right)\right|}_{\rho =0}>0.$$

Taking the derivative with respect to ρ at both sides of Equation (38), after some algebraic manipulations, we find that (56) is equivalent to

(57) $$\begin{array}{cc} \sum_{u_{\tau^{c}}}P_{\tau^{c}}\left(u_{\tau^{c}}\right)\sum_{x_{\tau^{c}},y}\sum_{u_{\tau },x_{\tau }} & P_{\tau |\tau^{c}}\left(u_{\tau }|u_{\tau^{c}}\right)Q_{\tau ,u_{\tau }}\left(x_{\tau }\right)Q_{\tau^{c},u_{\tau^{c}}}\left(x_{\tau^{c}}\right)W\left(y|x_{N}\right)\times \\ \; & \times \log\frac{P_{\tau |\tau^{c}}\left(u_{\tau }|u_{\tau^{c}}\right)Q_{\tau^{c},u_{\tau^{c}}}\left(x_{\tau^{c}}\right)W\left(y|x_{N}\right)}{\sum_{{\bar{u}}_{\tau },{\bar{x}}_{\tau }}P_{\tau |\tau^{c}}\left({\bar{u}}_{\tau }|u_{\tau^{c}}\right)Q_{\tau ,{\bar{u}}_{\tau }}\left({\bar{x}}_{\tau }\right)Q_{\tau^{c},u_{\tau^{c}}}\left(x_{\tau^{c}}\right)W\left(y|\left[{\bar{x}}_{\tau },x_{\tau^{c}}\right]\right)}>0. \end{array}$$

We next write the expression in the left hand-side of the inequality (57) in terms of entropy and mutual information. We denote as H(P) the entropy of a source with distribution *P* [32] (Eq. (2.1)) and by I(Q,W) the mutual information of a channel *W* with input distribution *Q* [32] (Eq. (2.28)). For $\sigma \subset 2^{N}$, we define a channel input distribution $Q_{\tau |\sigma }$, that is conditioned to the source messages $u_{\sigma }$, as

(58) $$Q_{\tau |\sigma }\left(x_{\tau }|u_{\sigma }\right)=\sum_{u_{\tau }\in U_{\tau }}P_{\tau |\sigma }\left(u_{\tau }|u_{\sigma }\right)Q_{\tau ,u_{\tau }}\left(x_{\tau }\right).$$

Therefore, the transmissibility condition (57) can be compactly expressed as

(59) $$\begin{array}{c} H\left(P_{\tau |\tau^{c}}\right)<I\left(Q_{\tau |\tau^{c}},W|P_{\tau^{c}}Q_{\tau^{c}|\tau^{c}}\right),\;\tau \subset 2^{N}\backslash \emptyset . \end{array}$$

As it is, $Q_{\tau^{c}|\tau^{c}}$ is “transparent”, as it cancels inside the fraction, and the channel law may also be written as $Q_{\tau^{c}|\tau^{c}}W$, removing the conditioning in the mutual information. With N={1,2} in Equation (59), we recover the achievable Cover-El Gamal-Salehi region [11] (Eq. (3)).

### 4.3. Numerical Examples

In this section, we present two simple examples showing that the exponent of the md-iid ensemble can be larger than that of the mi-iid ensemble with only two classes (and associated input distributions) for each user. First, we consider two correlated discrete memoryless sources, N=2 and N={1,2}, with alphabet $U_{\nu }=\left\{0,1\right\}$ for both users ν∈N, and probability distribution $P_{N}\left(u_{1},u_{2}\right)$ given in matrix form as

(60) $$P_{N}=\left(\begin{array}{cc} 0.0005 & 0.0095\\ 0.0005 & 0.9895 \end{array}\right).$$

The sources are sent over a discrete memoryless multiple-access channel with input alphabets $X_{1}=X_{2}=\left\{1,2,3,4,5,6\right\}$ and output alphabet Y={1,2,3,4}. The channel transition probabilites are given by a 36 × 4 matrix *W*, such that $W(y|x_{1},x_{2})$ is the row $x_{1}+6\left(x_{2}-1\right)$. The transition matrix *W* is given by

(61) $$\begin{array}{c} W=\left(\begin{array}{c} W_{1}\\ W_{2}\\ W_{3}\\ W_{4}\\ W_{5}\\ W_{6} \end{array}\right), \end{array}$$

where the 6 × 4 submatrices $W_{\ell }$, ℓ=1,…,6 are given as follows. First, the submatrix $W_{1}$ corresponds to the point-to-point channel discussed in ref. [8] (Sec. IV.C), given by

(62) $$\begin{array}{c} W_{1}=\left(\begin{array}{cccc} 1-3k_{1} & k_{1} & k_{1} & k_{1}\\ k_{1} & 1-3k_{1} & k_{1} & k_{1}\\ k_{1} & k_{1} & 1-3k_{1} & k_{1}\\ k_{1} & k_{1} & k_{1} & 1-3k_{1}\\ 0.5-k_{2} & 0.5-k_{2} & k_{2} & k_{2}\\ k_{2} & k_{2} & 0.5-k_{2} & 0.5-k_{2} \end{array}\right), \end{array}$$

for $k_{1}=0.045$ and $k_{2}=0.01$. Let the *m*-th row of matrix $W_{1}$ is denoted by $W_{1}\left(m\right)$. The matrix $W_{2}$ (resp. $W_{3}$) is a 6×4 matrix whose rows are all $W_{1}\left(5\right)$ (resp. $W_{1}\left(6\right)$). The matrices $W_{4}$, $W_{5}$ and $W_{6}$ are respectively given by

(63) $$\begin{array}{c} W_{4}=\left(\begin{array}{c} W_{1}\left(2\right)\\ W_{1}\left(3\right)\\ W_{1}\left(4\right)\\ W_{1}\left(1\right)\\ W_{1}\left(6\right)\\ W_{1}\left(5\right) \end{array}\right),\;W_{5}=\left(\begin{array}{c} W_{1}\left(3\right)\\ W_{1}\left(4\right)\\ W_{1}\left(1\right)\\ W_{1}\left(2\right)\\ W_{1}\left(5\right)\\ W_{1}\left(6\right) \end{array}\right),\;W_{6}=\left(\begin{array}{c} W_{1}\left(4\right)\\ W_{1}\left(1\right)\\ W_{1}\left(2\right)\\ W_{1}\left(3\right)\\ W_{1}\left(6\right)\\ W_{1}\left(5\right) \end{array}\right). \end{array}$$

The optimal achievable exponent [8] (Sec. IV.C) for the single-user channel $W_{1}$ in Equation (62) is related to two different distributions $Q^{☆}$ and $Q^{†}$, given in vector form by

(64) $$\begin{array}{c} Q^{☆}=\left(0,0,0,0,1\backslash 2,1\backslash 2\right), \end{array}$$

(65) $$\begin{array}{c} Q^{†}=\left(1\backslash 4,1\backslash 4,1\backslash 4,1\backslash 4,0,0\right). \end{array}$$

We let each user employ these distributions in the md-iid ensemble with input distribution in Equation (13) according to the source message partitioning in Equation (12) with $K_{\nu }=2$ classes per user and thresholds $\gamma_{N}=\left(\gamma_{1},\gamma_{2}\right)$. Since we consider two input distributions for each user, the channel Gallager function $\max_{\rho \in [0,1]}E_{0}\left(\rho ,Q_{\tau }^{i_{\tau }},WQ_{\tau^{c}}^{i_{\tau^{c}}}\right)$ is not concave in ρ [8]. To find the *md-iid* exponent $E^{\mathrm{md}-\mathrm{iid}}$, we optimize over the class thresholds following the method in Appendix B with the Gallager function in Equation (44), exploit the properties of the source function in Equation (45) in Appendix C, and also find the optimal input distribution assignment of $Q_{\nu }^{i_{\nu }}$ for each ν∈{1,2}. In our setting, we have four possible assignments, namely

(66) $$\begin{array}{c} \Omega_{1}\;:\;Q_{1}^{1}=Q_{2}^{1}=Q^{☆},\;Q_{1}^{2}=Q_{2}^{2}=Q^{†}, \end{array}$$

(67) $$\begin{array}{c} \Omega_{2}\;:\;Q_{1}^{1}=Q_{2}^{2}=Q^{☆},\;Q_{1}^{2}=Q_{2}^{1}=Q^{†}, \end{array}$$

(68) $$\begin{array}{c} \Omega_{3}\;:\;Q_{1}^{2}=Q_{2}^{1}=Q^{☆},\;Q_{1}^{1}=Q_{2}^{2}=Q^{†}, \end{array}$$

(69) $$\begin{array}{c} \Omega_{4}\;:\;Q_{1}^{2}=Q_{2}^{2}=Q^{☆},\;Q_{1}^{1}=Q_{2}^{1}=Q^{†}. \end{array}$$

**Figure 2:**
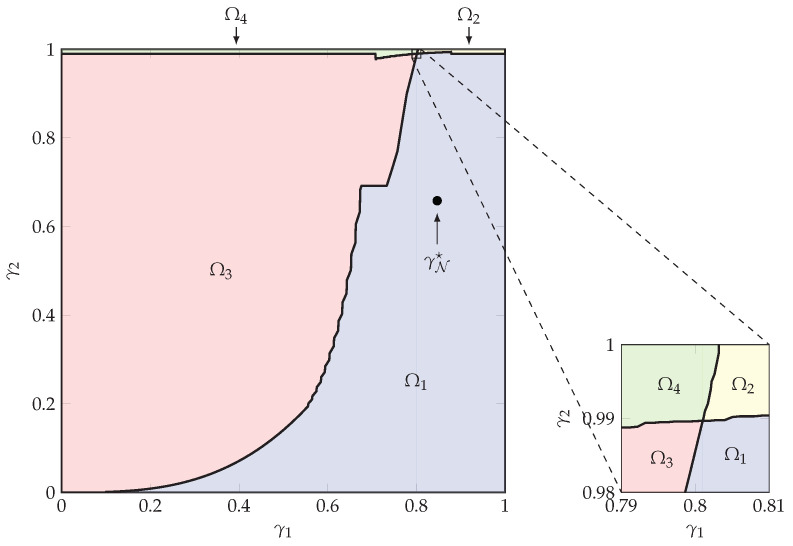
Correlated-sources optimal assignment $\Omega^{☆}\left(\gamma_{N}\right)$ in Equation (70) for all pairs of thresholds $\left(\gamma_{1},\gamma_{2}\right)$.

**Figure 3:**
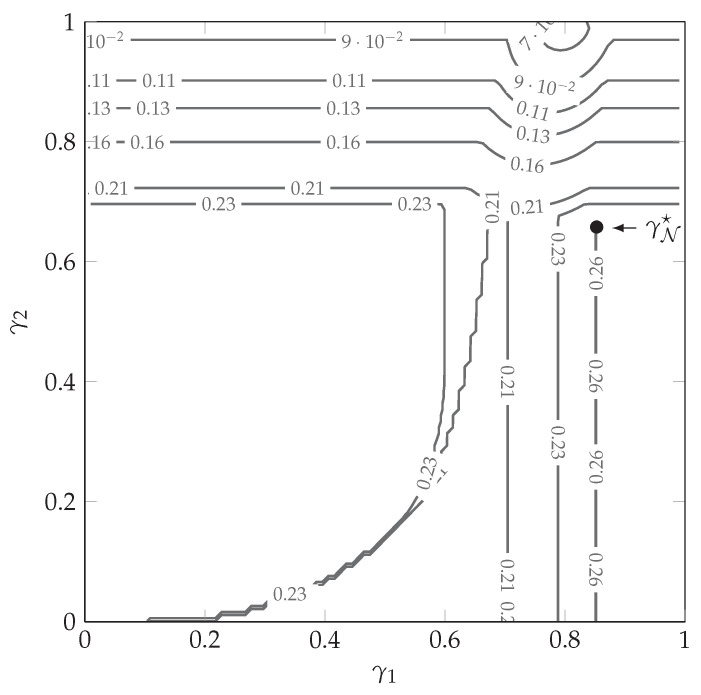
Correlated-sources error exponent $E^{cost}\left(\gamma_{N}\right)$ in Equation (71) for all pairs of thresholds $\left(\gamma_{1},\gamma_{2}\right)$.

We start our numerical discussion by assessing which of the possible four assignments in Equations (66)–(69) leads to a higher error exponent. For each possible pair of thresholds $\left(\gamma_{1},\gamma_{2}\right)$, we numerically calculate the optimal assignment $\Omega^{☆}\left(\gamma_{N}\right)$ given by

(70) $$\Omega^{☆}\left(\gamma_{N}\right)=\underset{\Omega_{j}}{arg max}\min_{i_{N}}\min_{\tau }E_{\tau }^{i_{N}}\left(\gamma_{N}\right),$$

and the corresponding achievable error exponent $E^{md-iid}\left(\gamma_{N}\right)$ as

(71) $$E^{cost}\left(\gamma_{N}\right)=\max_{\Omega_{j}}\min_{i_{N}}\min_{\tau }E_{\tau }^{i_{N}}\left(\gamma_{N}\right),$$

where the exponent function $E_{\tau }^{i_{N}}\left(\gamma_{N}\right)$ is given in Equation (A55). Figure 2 and Figure 3 respectively show $\Omega^{☆}\left(\gamma_{N}\right)$ and $E^{cost}\left(\gamma_{N}\right)$ for the valid range of $\gamma_{N}$. For most pair of thresholds $\left(\gamma_{1},\gamma_{2}\right)$, assignments $\Omega_{1}$ and $\Omega_{3}$ lead to the highest exponent among the possible assignments, while assignments $\Omega_{2}$ and $\Omega_{4}$ are optimal only for a marginal region. Using this information, and combined with the values of the achievable exponents in Figure 3, we determine the message-dependent exponent

(72) $$E^{md-iid}=\max_{\gamma_{N}}E^{cost}\left(\gamma_{N}\right).$$

In this example, we obtained the achievable exponent $E^{\mathrm{md}-\mathrm{iid}}=0.2611$, corresponding to the input distribution assignment $\Omega_{1}$ in Equation (66) and optimal source message partitioning $\gamma_{1}^{☆}=0.8469$ and $\gamma_{2}^{☆}=0.6581$. The optimal point $\gamma_{N}^{☆}$ is shown by a white (black) bullet in Figure 2 (Figure 3).

Alternatively, we may first optimize over $\gamma_{N}$ and then over the assignments $\Omega_{j}$. To do so, we solve the system of Equation (A58) in Appendix B to numerically determine the optimal thresholds $\gamma_{N}^{☆}$, and compute the exponent $E^{\mathrm{cost}}\left(\Omega_{j}\right)$ as

(73) $$E^{\mathrm{cost}}\left(\Omega_{j}\right)=\min_{i_{N}}\min_{\tau }E_{\tau }^{i_{N}}\left(\gamma_{N}^{☆}\right),$$

where the exponent function $E_{\tau }^{i_{N}}\left(\gamma_{N}\right)$ is given in Equation (A55). We provide in Table 1 the values of the optimal thresholds $\gamma_{N}^{☆}$ and exponents $E_{\tau }^{i_{N}}\left(\gamma_{N}^{☆}\right)$ under the different assignment $\Omega_{j}$, for the three types of error τ and the four possible user classes $i_{N}$. For each assignment, the minimum over $i_{N}$ and τ as in Equation (73) is highlighted in gray, leading to the exponent $E^{\cos t}\left(\Omega_{j}\right)$. The message-dependent exponent is then

(74) $$\begin{array}{c} E^{\mathrm{md}-\mathrm{iid}}=\max_{j}E^{\cos t}\left(\Omega_{j}\right), \end{array}$$

recovering the error exponent $E^{\mathrm{md}-\mathrm{iid}}=0.2611$ for input distribution assignment $\Omega_{1}$ obtained using the previous method in Equation (71).

In the second example, we consider the transmission of two independent discrete memoryless sources with identical source alphabets $U_{\nu }=\left\{0,1\right\}$ with distributions induced by the marginals of Equation (60), given by $P_{1}\left(0\right)=0.01$ and $P_{2}\left(0\right)=0.001$. These sources are transmitted over the multiple-access channel with transition probability given by Equation (61), and are encoded using the md-iid ensemble with the input distribution assignments $\Omega_{j}$ in Equations (66)–(69). Following the footsteps of the correlated sources case, in Table 2 we calculate optimal thresholds $\gamma_{N}^{☆}$ and exponents $E_{\tau }^{i_{N}}\left(\gamma_{N}^{☆}\right)$ for the possible input distribution assignments and determine the exponent of the md-iid ensemble using Equations (73) and (74). In this case, the optimal assignment is again $\Omega_{1}$, with optimal source message partitioning specified by the thresholds $\gamma_{1}^{☆}=0.8779$ and $\gamma_{2}^{☆}=0.6933$, achieving an exponent of $E^{\mathrm{md}-\mathrm{iid}}=0.2458$, slightly smaller than that of correlated sources.

For the sake of completeness and purpose of comparison, we also calculate the exponent for the *mi-iid* ensemble described in Equation (8). In the absence of message dependence, for a given assignment $\Omega_{j}$, the *mi-iid* exponent is given by

(75) $$\begin{array}{c} E^{\mathrm{no}-\mathrm{cost}}\left(\Omega_{j}\right)=\min_{\tau }E_{\tau }, \end{array}$$

where the exponent function $E_{\tau }$ is given by $E_{\tau }=\max_{\rho }E_{\tau }^{\mathrm{mi}-\mathrm{iid}}\left(\rho ,P_{N},Q_{N},W\right)$ and $E_{\tau }^{\mathrm{mi}-\mathrm{iid}}$ is the Gallager function in Equation (37), described in the previous subsection. For both the correlated and independent sources described above, Table 3 presents the achievable exponents $E_{\tau }$ for each type of error τ and input distribution assignment $\left(Q_{1},Q_{2}\right)$, where $Q_{1}$ and $Q_{2}$ are either of $Q^{☆}$ and $Q^{†}$ in Equations (64) and (65). In our numerical example for correlated sources, the assignment with highest exponent is $\left(Q_{1},Q_{2}\right)=\left(Q^{†},Q^{☆}\right)$, giving an exponent of $E^{\mathrm{mi}-\mathrm{iid}}=0.2503$, slightly smaller than that of the md-iid ensemble. In contrast, the mi-iid exponent for independent sources, according to the second part of Table 3 is found to be $E^{\mathrm{mi}-\mathrm{iid}}=0.2367$ with input distribution $\left(Q_{1},Q_{2}\right)=\left(Q^{☆},Q^{†}\right)$. In this case, the md-iid exponent $E^{\mathrm{md}-\mathrm{iid}}$ is around 4% larger that the *mi-iid*; this situation is in contrast with to-point communication, where the gain in exponent achieved by an ensemble with two distributions is typically smaller, for example, 1% in ref. [8]. Hence, message-dependent random coding with two class distributions, compared to iid random coding, may lead to a higher error exponent gain in the MAC than in point-to-point communication.

### 4.4. Comparison of the Random-Coding Achievable Error Exponents

From the numerical results presented in Section 4.3, as well as from refs. [8,20,28,31], the message-dependent ensembles attain in general a larger exponent than their message-independent counterparts. We now compare the random-coding exponents for the ensembles presented in Section 3.1, whose Gallager functions were obtained in Section 4.1.

For independent sources, we found in Equation (42) that for a given conditional input distribution $Q_{\nu ,u_{\nu }}\left(x_{\nu }\right)$ and ρ, there exists an *iiid* distribution $Q_{\nu ,\rho }$ given by Equation (39) with identical Gallager function. Thus, the *mi-iid* and *mi-icd* ensembles attains the same exponent, after maximization over the input distributions. Similarly, we conclude that *md-iid* and *md-icd*-ensembles attain the same exponent.

In ref. [31] (Prop. 2.9), it was proved that for point-to-point communication, the exponent of the mi-ccc ensemble may be lower than that of the mi-cc ensemble. The same steps actually prove the same result for the MAC with independent sources. Thus, for the MAC with independent sources we have

(76) $$\begin{array}{c} E^{mi-ccc}\leq E^{mi-cc}\leq E^{md-cc},\;E^{md-ccc}\leq E^{md-cc}, \end{array}$$

(77) $$\begin{array}{c} E^{mi-iid}\leq E^{mi-cc}\leq E^{md-cc},\;E^{md-iid}\leq E^{md-cc}, \end{array}$$

and $E^{md-cc}$ is thus largest among the ensembles in Section 3.1 for an arbitrary input distribution. As discussed in ref. [29] (Th. 4), for optimal input distributions both $E^{md-cc}$ and $E^{md-iid}$ may coincide.

Concerning the optimal partitioning into message classes, for point-to-point communication it is known that partitioning the source-message set into two classes is sufficient to attain the optimal error exponent [8,31] (Prop. 2.7). However, the proof of ref. [31] (Prop. 2.7) cannot be easily generalized to the MAC with independent sources. At the same time, we could not find an example showing that assigning more than two input distributions leads to a larger exponent. Hence, finding the sufficient number of input distributions is for the message-dependent exponent is an open problem.

The comparisons in Equations (76) and (77) for correlated sources require, in general, a more sophisticated machinery and we consider here two simple cases. For the message-dependent *md-icd* and *md-ccc* ensembles, we observe that compared to $E_{\tau ,i_{N}}^{md-icd}$ in Equation (43) the $E_{\tau ,i_{N}}^{md-ccc}$ exponent in Equation (54) contains an additional term $\beta_{\tau ,N,u_{N}}^{i_{N}}\left(x_{N}\right)$ to guarantee the constant-composition distribution as in Equation (52). This allows to recover $E_{\tau ,i_{N}}^{md-ccc}$ by setting $\beta_{\tau ,N,u_{N}}^{i_{N}}\left(x_{N}\right)=0$ in $E_{\tau ,i_{N}}^{md-icd}$ and to prove that $E^{md-icd}\leq E^{md-ccc}$ after maximizing w. r. t. $\beta_{\tau ,N,u_{N}}^{i_{N}}(x_{N})$. Similarly for the ensembles with statistical independence between messages and codewords, we observe that the constant-composition exponent $E_{\tau ,i_{N}}^{md-cc}$ in Equation (51) also contains the additional term $\alpha_{\tau ,N}^{i_{N}}\left(x_{N}\right)$ compared to its *iid* counterpart $E_{\tau ,i_{N}}^{md-iid}$ in Equation (44), yielding $E^{md-iid}\leq E^{md-cc}$. Put together, for correlated sources it holds that

(78) $$\begin{array}{c} E^{md-icd}\leq E^{md-ccc},\;E^{md-iid}\leq E^{md-cc}, \end{array}$$

suggesting that, as in the case of single-user communication, the use of constant-composition input distributions may lead to higher exponents than the symbol-wise independent distributions when transmitting correlated sources over the MAC.

Summarizing, proper choices of the cost functions recover the different coding schemes considered in Section 3.1, including message-dependent and message-independent versions of iid, independent conditionally distributed, constant-composition, and conditional constant composition ensembles. Thanks to the flexibility of the generalized cost-constraint random-coding ensemble, the achievable exponents of the various ensembles can be compared and ranked, both numerically and analytically. 

## Figures and Tables

**Table 1:** Correlated-sources optimal thresholds $\gamma_{N}^{☆}$ and exponents $E_{\tau }^{i_{N}}\left(\gamma_{N}^{☆}\right)$ in Equation (73) for assignments $\Omega_{j}$ in Equations (66)–(69). For each assignment, the minimum over $i_{N}$ and τ is highlighted in gray.

	Assignment $\Omega_{1}$					Assignment $\Omega_{2}$			
	$\gamma_{1}^{☆}=0.8469$ $\gamma_{2}^{☆}=0.6581$					$\gamma_{1}^{☆}=1$ $\gamma_{2}^{☆}=1$			
$\left(i_{1},i_{2}\right)$	(1,1)	(1,2)	(2,1)	(2,2)		(1,1)	(1,2)	(2,1)	(2,2)
τ={1}	0.3131	0.2735	0.3120	0.2611		0.0642	0.3268	0.1005	0.3604
τ={2}	0.3986	0.4369	0.2611	0.4119		0.3959	0.3986	0.4323	0.3110
τ={1,2}	0.2611	0.2972	0.2630	0.2883		0.2108	0.2108	0.2360	0.2637
	**Assignment** $\Omega_{3}$					**Assignment** $\Omega_{4}$			
	$\gamma_{1}^{☆}=0.5605$ $\gamma_{2}^{☆}=0.6709$					$\gamma_{1}^{☆}=0.6985$ $\gamma_{2}^{☆}=0.9033$			
$\left(i_{1},i_{2}\right)$	(1,1)	(1,2)	(2,1)	(2,2)		(1,1)	(1,2)	(2,1)	(2,2)
τ={1}	0.3120	0.2503	0.2763	0.2897		0.0879	0.3605	0.0879	0.3112
τ={2}	0.2503	0.3898	0.5675	0.5731		0.3664	0.2503	0.4720	0.4684
τ={1,2}	0.2630	0.2816	0.2503	0.3012		0.2360	0.2632	0.2097	0.2097

**Table 2:** Independent-sources md-iid optimal thresholds $\gamma_{N}^{☆}$ and exponents $E_{\tau }^{i_{N}}\left(\gamma_{N}^{☆}\right)$ in Equation (73) for assignments $\Omega_{j}$ in Equations (66)–(69). For each assignment, the minimum over $i_{N}$ and τ is highlighted in gray.

	Assignment $\Omega_{1}$					Assignment $\Omega_{2}$			
	$\gamma_{1}^{☆}=0.8779$ $\gamma_{2}^{☆}=0.6933$					$\gamma_{1}^{☆}=0.8776\;$ $\gamma_{2}^{☆}=1$			
$\left(i_{1},i_{2}\right)$	(1,1)	(1,2)	(2,1)	(2,2)		(1,1)	(1,2)	(2,1)	(2,2)
τ={1}	0.3343	0.2458	0.3089	0.2458		0.0913	0.3341	0.0913	0.3089
τ={2}	0.3850	0.3987	0.2458	0.3788		0.4555	0.3850	0.4357	0.2459
τ={1,2}	0.2730	0.2870	0.2685	0.2863		0.3430	0.2728	0.2956	0.2685
	**Assignment** $\Omega_{3}$					**Assignment** $\Omega_{4}$			
	$\gamma_{1}^{☆}=0.61$ $\gamma_{2}^{☆}=0.7043$					$\gamma_{1}^{☆}=0.7092$ $\gamma_{2}^{☆}=1$			
$\left(i_{1},i_{2}\right)$	(1,1)	(1,2)	(2,1)	(2,2)		(1,1)	(1,2)	(2,1)	(2,2)
τ={1}	0.3089	0.2367	0.2681	0.3078		0.0913	0.3117	0.0913	0.2648
τ={2}	0.2367	0.3672	0.5425	0.5538		0.4269	0.2367	0.5393	0.4683
τ={1,2}	0.2685	0.2811	0.2367	0.3133		0.3006	0.2685	0.2740	0.2164

**Table 3:** Mi-iid exponents $E_{\tau }$ in Equation (75) for two correlated and two independent sources vs several input distribution assigments $\left(Q_{1},Q_{2}\right)$. For each assignment, the minimum over τ is highlighted in gray.

	Correlated Sources			
$\left(Q_{1},Q_{2}\right)$	$\left(Q^{☆},Q^{☆}\right)$	$\left(Q^{☆},Q^{†}\right)$	$\left(Q^{†},Q^{☆}\right)$	$\left(Q^{†},Q^{†}\right)$
τ={1}	0.2682	0.0642	0.3120	0.0879
τ={2}	0.3986	0.3986	0.2503	0.3696
τ={1,2}	0.2097	0.2097	0.2630	0.2360
	**Independent Sources**			
$\left(Q_{1},Q_{2}\right)$	$\left(Q^{☆},Q^{☆}\right)$	$\left(Q^{☆},Q^{†}\right)$	$\left(Q^{†},Q^{☆}\right)$	$\left(Q^{†},Q^{†}\right)$
τ={1}	0.2648	0.3089	0.0627	0.0865
τ={2}	0.3850	0.2367	0.3850	0.3559
τ={1,2}	0.2164	0.2685	0.2164	0.2421

**Figure A1:**
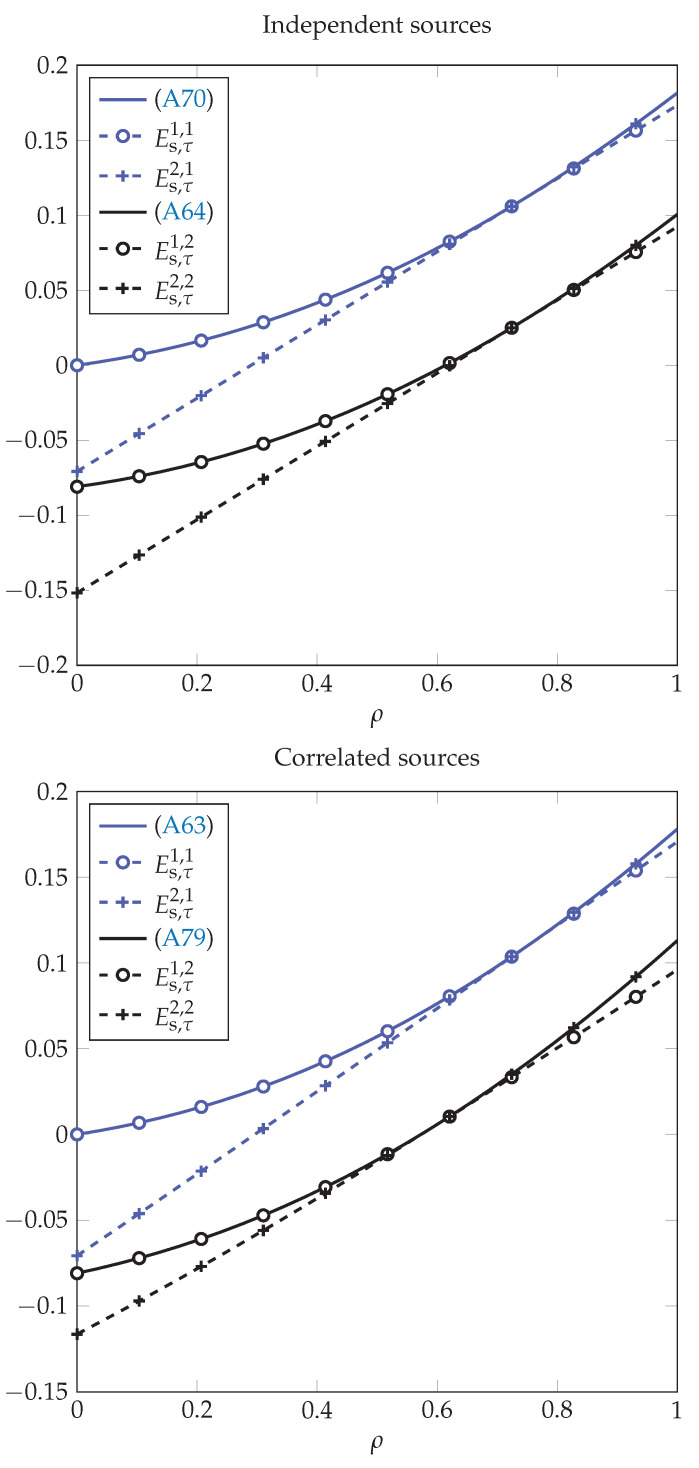
Example of the source functions $E_{s,\tau }^{i_{N}}$ in Equation (A62) for independent and correlated sources and error type $\tau \in \left\{\{1\},\{2\}\right\}$.

## Data Availability

Data is contained within the article.

## Appendix A. Proof of Theorem 1

We start by bounding the average error probability over the generalized cost-constraint ensemble, ${\bar{P}}_{e}$. Counting ties as errors, the random coding union bound [2] (Th. 16) for joint source-channel is

(A1) $$\begin{array}{c} {\bar{P}}_{e}\leq \sum_{u_{N},x_{N},y}P_{N}\left(u_{N}\right)Q_{N}\left(x_{N}|u_{N}\right)W\left(y|x_{N}\right)\min\left\{1,\sum_{\begin{array}{c} {\hat{u}}_{N}\neq u_{N} \end{array}}\mathrm{Pr}\left\{\frac{P_{N}\left({\hat{u}}_{N}\right)W\left(y|{\hat{X}}_{N}\right)}{P_{N}\left(u_{N}\right)W\left(y|x_{N}\right)}\geq 1\right\}\right\}, \end{array}$$

where $Q_{N}\left(x_{N}|u_{N}\right)$ is given by Equation (7), with every user using the generalized cost-constrained input distribution $Q_{\nu }\left(x_{\nu }|u_{\nu }\right)$ as in Equation (24), and ${\hat{x}}_{N}$ has the same distribution as $x_{N}$ but conditioned on ${\hat{u}}_{N}$ rather than $u_{N}$, i.e., $Q_{N}\left(x_{N}|{\hat{u}}_{N}\right)$. The summation over ${\hat{u}}_{N}\neq u_{N}$ can be split into $2^{N}-1$ distinct types of error events indexed by the non-empty subsets in the power set of the user indices $2^{N}\backslash \emptyset$, e.g., τ∈{{1},{2},{1,2}} for N=2, such that ${\hat{u}}_{\tau^{c}}=u_{\tau^{c}}$ and ${\hat{u}}_{\nu }\neq u_{\nu }$ for all ν∈τ.

Since min{1,a+b}≤min{1,a}+min{1,b}, we bound ${\bar{P}}_{e}$ as

(A2) $$\begin{array}{c} {\bar{P}}_{e}\leq \sum_{\tau \in 2^{N}\backslash \emptyset }{\bar{P}}_{e}^{\tau }, \end{array}$$

where ${\bar{P}}_{e}^{\tau }$ is in turn given by

(A3) $$\begin{array}{c} \;{\bar{P}}_{e}^{\tau }=\;\sum_{u_{N},x_{N},y}\;P_{N}\left(u_{N}\right)Q_{N}\left(x_{N}|u_{N}\right)W\left(y|x_{N}\right)\min\left\{1,\sum_{\begin{array}{c} {\hat{u}}_{N}\;:\;{\hat{u}}_{\tau^{c}}=u_{\tau^{c}},\\ {\hat{u}}_{\nu }\neq u_{\nu },\nu \in \tau \end{array}}\mathrm{Pr}\left\{\frac{P_{N}\left({\hat{u}}_{N}\right)W\left(y|[x_{\tau^{c}},{\hat{X}}_{\tau }]\right)}{P_{N}\left(u_{N}\right)W\left(y|x_{N}\right)}\geq 1\right\}\right\}, \end{array}$$

where the inner probability is computed according to the distribution $Q_{\tau }\left(x_{\tau }|u_{\tau }\right)$, including only users $u_{\tau }$ in the set τ as ${\hat{x}}_{\tau^{c}}=x_{\tau^{c}}$. We recall that $\left[x_{\tau^{c}},{\hat{X}}_{\tau }\right]$ is the sorted merger of the channel inputs for users in the sets $\tau^{c}$ and τ, in this case $x_{\tau^{c}}$ and ${\hat{X}}_{\tau }$ respectively.

Next, we split the summation over $u_{N}$ in Equation (A3) into classes $i_{N}\in K_{N}$ defined by Equation (12), summing then over the messages belonging to the Cartesian product of the sets $A_{N}^{i_{N}}$. We note that codewords are generated according to distributions that depend on the class index of the sources. Let $D_{N,u_{N}}^{i_{N}}$ be the Cartesian product of the sets of codewords $D_{\nu ,u_{\nu }}^{i_{\nu }}$ in Equation (23) for ν=1,2,…,N, and define

(A4) $$\begin{array}{c} Q_{N}^{i_{N}}\left(x_{N}|u_{N}\right)=\prod_{\nu \in N}Q_{\nu }^{i_{\nu }}\left(x_{\nu }|u_{\nu }\right), \end{array}$$

where $Q_{\nu }^{i_{\nu }}\left(x_{\nu }|u_{\nu }\right)$ is given by either Equation (24) or Equation (25). Then, the double outer summation of Equation (A3) over $u_{N}$ and $x_{N}$ can be written as

(A5) $$\begin{array}{cc} \sum_{u_{N},x_{N}} & P_{N}\left(u_{N}\right)Q_{N}\left(x_{N}|u_{N}\right)=\sum_{i_{N}\in K_{N}}\sum_{\begin{array}{c} u_{N}\in A_{N}^{i_{N}}\\ x_{N}\in D_{N,u_{N}}^{i_{N}} \end{array}}P_{N}\left(u_{N}\right)Q_{N}^{i_{N}}\left(x_{N}|u_{N}\right) \end{array}$$

(A6) $$\begin{array}{cc} \; & =\sum_{i_{\tau^{c}}\in K_{\tau^{c}}}\sum_{\begin{array}{c} u_{\tau^{c}}\in A_{\tau^{c}}^{i_{\tau^{c}}}\\ x_{\tau^{c}}\in D_{\tau^{c},u_{\tau^{c}}}^{i_{\tau^{c}}} \end{array}}P_{\tau^{c}}\left(u_{\tau^{c}}\right)Q_{\tau^{c}}^{i_{\tau^{c}}}\left(x_{\tau^{c}}|u_{\tau^{c}}\right)\sum_{i_{\tau }\in K_{\tau }}\sum_{\begin{array}{c} u_{\tau }\in A_{\tau }^{i_{\tau }}\\ x_{\tau }\in D_{\tau ,u_{\tau }}^{i_{\tau }} \end{array}}P_{\tau |\tau^{c}}\left(u_{\tau }|u_{\tau^{c}}\right)Q_{\tau }^{i_{\tau }}\left(x_{\tau }|u_{\tau }\right), \end{array}$$

where we split the summations over $u_{N}$ and $x_{N}$ into separate summations over $u_{\tau^{c}}$ and $u_{\tau }$, similarly with $x_{\tau^{c}}$ and $x_{\tau }$ with the corresponding rearrangements in the probabilities, and written the term $Q_{\tau^{c}}^{i_{\tau^{c}}}\left(x_{\tau^{c}}|u_{\tau^{c}}\right)$ in a similar way to Equation (A4). The inner summation of Equation (A3) can be split in an analogous manner based on the classes to which ${\hat{u}}_{\tau }$ belongs, now indexed by the variable $j_{\tau }\in K_{\tau }$. Applying this fact together with Markov’s inequality

(A7) $$\begin{array}{c} \mathrm{Pr}\left\{A\geq 1\right\}\leq \min_{s\geq 0}\;E\left[A^{s}\right] \end{array}$$

to upper bound the probability with a parameter s≥0 that implicitly depends on the error-event type τ and indices $i_{\tau^{c}}$, $i_{\tau }$, and $j_{\tau }$. We bound the inner summation of Equation (A3) as

(A8) $$\begin{array}{cc} \sum_{\begin{array}{c} {\hat{u}}_{N}\;:\;{\hat{u}}_{\tau^{c}}=u_{\tau^{c}},\\ {\hat{u}}_{\nu }\neq u_{\nu },\nu \in \tau \end{array}} & \mathrm{Pr}\left\{\frac{P_{N}\left({\hat{u}}_{N}\right)W\left(y|[x_{\tau^{c}},{\hat{X}}_{\tau }]\right)}{P_{N}\left(u_{N}\right)W\left(y|x_{N}\right)}\geq 1\right\}\leq \\ \; & \leq \sum_{j_{\tau }\in K_{\tau }}\min_{s\geq 0}\left\{\sum_{\begin{array}{c} {\hat{u}}_{\tau }\in A_{\tau }^{j_{\tau }}\\ {\hat{x}}_{\tau }\in D_{\tau ,{\hat{u}}_{\tau }}^{j_{\tau }} \end{array}}Q_{\tau }^{j_{\tau }}\left({\hat{x}}_{\tau }|{\hat{u}}_{\tau }\right){\left(\frac{P_{\tau |\tau^{c}}\left({\hat{u}}_{\tau }|u_{\tau^{c}}\right)W\left(y|[x_{\tau^{c}},{\hat{x}}_{\tau }]\right)}{P_{\tau |\tau^{c}}\left(u_{\tau }|u_{\tau^{c}}\right)W\left(y|[x_{\tau^{c}},x_{\tau }]\right)}\right)}^{s}\right\}, \end{array}$$

where we also used that $P_{N}\left({\hat{u}}_{N}\right)=P_{\tau^{c}}\left({\hat{u}}_{\tau^{c}}\right)P_{\tau |\tau^{c}}\left({\hat{u}}_{\tau }|{\hat{u}}_{\tau^{c}}\right)=P_{\tau^{c}}\left(u_{\tau^{c}}\right)P_{\tau |\tau^{c}}\left({\hat{u}}_{\tau }|u_{\tau^{c}}\right)$ to rewrite the message probabilities in Equation (A8) and we expressed the codeword $x_{N}$ as $\left[x_{\tau^{c}},x_{\tau }\right]$.

Inserting Equations (A6) and (A8) into Equation (A3) and using the following inequality for A≥0,

(A9) $$\begin{array}{c} \min\left\{1,A\right\}\leq \min_{\rho \in [0,1]}A^{\rho }, \end{array}$$

where ρ∈[0,1], we further bound ${\bar{P}}_{e}^{\tau }$ as

(A10) $$\begin{array}{c} {\bar{P}}_{e}^{\tau }\leq \sum_{i_{\tau^{c}}\in K_{\tau^{c}}}\sum_{i_{\tau }\in K_{\tau }}\sum_{j_{\tau }\in K_{\tau }}\min_{s\geq 0}\min_{\rho \in [0,1]}{\bar{P}}_{e,i_{\tau^{c}}i_{\tau }}^{\tau ,j_{\tau }}, \end{array}$$

where after some minor rearrangements ${\bar{P}}_{e,i_{\tau^{c}}i_{\tau }}^{\tau ,j_{\tau }}$ is in turn given by

(A11) $$\begin{array}{cc} {\bar{P}}_{e,i_{\tau^{c}}i_{\tau }}^{\tau ,j_{\tau }}=\sum_{\begin{array}{c} u_{\tau^{c}}\in A_{\tau^{c}}^{i_{\tau^{c}}}\\ x_{\tau^{c}}\in D_{\tau^{c},u_{\tau^{c}}}^{i_{\tau^{c}}} \end{array}}\; & P_{\tau^{c}}\left(u_{\tau^{c}}\right)Q_{\tau^{c}}^{i_{\tau^{c}}}\left(x_{\tau^{c}}|u_{\tau^{c}}\right)\sum_{y\in Y^{n}}\sum_{\begin{array}{c} u_{\tau }\in A_{\tau }^{i_{\tau }}\\ x_{\tau }\in D_{\tau ,u_{\tau }}^{i_{\tau }} \end{array}}P_{\tau |\tau^{c}}\left(u_{\tau }|u_{\tau^{c}}\right)Q_{\tau }^{i_{\tau }}\left(x_{\tau }|u_{\tau }\right)W\left(y|[x_{\tau^{c}},x_{\tau }]\right)\\ \; & {\left(\sum_{\begin{array}{c} {\hat{u}}_{\tau }\in A_{\tau }^{j_{\tau }}\\ {\hat{x}}_{\tau }\in D_{\tau ,{\hat{u}}_{\tau }}^{j_{\tau }} \end{array}}Q_{\tau }^{j_{\tau }}\left({\hat{x}}_{\tau }|{\hat{u}}_{\tau }\right){\left(\frac{P_{\tau |\tau^{c}}\left({\hat{u}}_{\tau }|u_{\tau^{c}}\right)W\left(y|[x_{\tau^{c}},{\hat{x}}_{\tau }]\right)}{P_{\tau |\tau^{c}}\left(u_{\tau }|u_{\tau^{c}}\right)W\left(y|[x_{\tau^{c}},x_{\tau }]\right)}\right)}^{s}\right)}^{\rho }. \end{array}$$

Note that, for some conveniently chosen variables $z_{0}$ and $z_{i_{\tau }}$, sets $Z_{0}$ and $Z_{i_{\tau }}$, as well as functions $f_{0}\left(z_{0}\right)$ and $f_{i_{\tau }}^{s}\left(z_{0},z_{i_{\tau }}\right)$, with $i_{\tau }\in K_{\tau }$, we can express ${\bar{P}}_{e,i_{\tau^{c}}i_{\tau }}^{\tau ,j_{\tau }}$ as

(A12) $$\begin{array}{c} {\bar{P}}_{e,i_{\tau^{c}}i_{\tau }}^{\tau ,j_{\tau }}=\sum_{z_{0}\in Z_{0}}f_{0}\left(z_{0}\right)\left(\sum_{z_{i_{\tau }}\in Z_{i_{\tau }}}f_{i_{\tau }}^{1-s_{i_{\tau },j_{\tau }}\rho_{i_{\tau },j_{\tau }}}\left(z_{0},z_{i_{\tau }}\right)\right){\left(\sum_{z_{j_{\tau }}\in Z_{j_{\tau }}}f_{j_{\tau }}^{s_{i_{\tau },j_{\tau }}}\left(z_{0},z_{j_{\tau }}\right)\right)}^{\rho_{i_{\tau },j_{\tau }}}. \end{array}$$

In Equation (A12), the variable $z_{0}$ stands for the triplet $\left(u_{\tau^{c}},x_{\tau^{c}},y\right)$, the alphabet $Z_{0}$ for the Cartesian product $A_{\tau^{c}}^{i_{\tau^{c}}}\times D_{\tau^{c},u_{\tau^{c}}}^{i_{\tau^{c}}}\times Y^{n}$ and the function $f_{0}\left(z_{0}\right)$ is given by $P_{\tau^{c}}\left(u_{\tau^{c}}\right)Q_{\tau^{c}}^{i_{\tau^{c}}}\left(x_{\tau^{c}}|u_{\tau^{c}}\right)$. The variable $z_{i_{\tau }}$ stands for the pair $\left(u_{\tau },x_{\tau }\right)$, the alphabet $Z_{i_{\tau }}$ for the Cartesian product $A_{\tau }^{i_{\tau }}\times D_{\tau ,u_{\tau }}^{i_{\tau }}$ and the function $f_{i_{\tau }}^{s}\left(z_{0},z_{i_{\tau }}\right)$ is given by $P_{\tau |\tau^{c}}{\left(u_{\tau }|u_{\tau^{c}}\right)}^{s}Q_{\tau }^{i_{\tau }}\left(x_{\tau }|u_{\tau }\right)W{\left(y|[x_{\tau^{c}},x_{\tau }]\right)}^{s}$.

The optimization parameters *s* and ρ in Equation (A10) implictly depend on the error-event type τ and the indices $i_{\tau^{c}}$, $i_{\tau }$, and $j_{\tau }$. For new parameters ${\bar{\rho }}_{\ell_{\tau }}\in \left[0,1\right]$, $\ell_{\tau }\in K_{\tau }$, setting

(A13) $$\begin{array}{c} s_{i_{\tau },j_{\tau }}=\frac{1}{1+{\bar{\rho }}_{j_{\tau }}}, \end{array}$$

(A14) $$\begin{array}{c} \rho_{i_{\tau },j_{\tau }}=\frac{{\bar{\rho }}_{i_{\tau }}\left(1+{\bar{\rho }}_{j_{\tau }}\right)}{1+{\bar{\rho }}_{i_{\tau }}}, \end{array}$$

In Equation (A12), we obtain the following partial upper bound in Equation (A10) as

(A15) $$\begin{array}{c} \;\min_{s\geq 0}\min_{\rho \in [0,1]}{\bar{P}}_{e,i_{\tau^{c}}i_{\tau }}^{\tau ,j_{\tau }}\leq \min_{{\bar{\rho }}_{\ell_{\tau }}\in {\left[0,1\right]}^{K_{\tau }}}\sum_{z_{0}\in Z_{0}}f_{0}\left(z_{0}\right)\sum_{z_{i_{\tau }}\in Z_{i_{\tau }}}f_{i_{\tau }}^{\frac{1}{1+{\bar{\rho }}_{i_{\tau }}}}\left(z_{0},z_{i_{\tau }}\right){\left(\sum_{z_{j_{\tau }}\in Z_{j_{\tau }}}f_{j_{\tau }}^{\frac{1}{1+{\bar{\rho }}_{j_{\tau }}}}\left(z_{0},z_{j_{\tau }}\right)\right)}^{\frac{{\bar{\rho }}_{i_{\tau }}\left(1+{\bar{\rho }}_{j_{\tau }}\right)}{1+{\bar{\rho }}_{i_{\tau }}}}. \end{array}$$

Here, we have kept implicit the dependence on τ and $i_{\tau^{c}}$ of the optimization parameter ${\bar{\rho }}_{\ell_{\tau }}$. Now, applying Hölder’s inequality [33] (Th. 13) in the form

(A16) $$\begin{array}{c} \sum_{i\in K}\alpha_{i}a_{i}b_{i}\leq {\left(\sum_{i\in K}\alpha_{i}a_{i}^{p}\right)}^{\frac{1}{p}}{\left(\sum_{i\in K}\alpha_{i}b_{i}^{\frac{p}{p-1}}\right)}^{\frac{p-1}{p}},\;\mathrm{for}p\in \left[1,\infty \right), \end{array}$$

to the expression in Equation (A15) with $p_{i_{\tau },j_{\tau }}=1+{\bar{\rho }}_{i_{\tau }}\geq 1$, we obtain

(A17) $$\begin{array}{cc} \min_{s\geq 0}\min_{\rho \in [0,1]}{\bar{P}}_{e,i_{\tau^{c}}i_{\tau }}^{\tau ,j_{\tau }}\leq \min_{{\bar{\rho }}_{\ell_{\tau }}\in {\left[0,1\right]}^{K_{\tau }}} & {\left(\sum_{z_{0}\in Z_{0}}f_{0}\left(z_{0}\right){\left(\sum_{z_{i_{\tau }}\in Z_{i_{\tau }}}f_{i_{\tau }}^{\frac{1}{1+{\bar{\rho }}_{i_{\tau }}}}\left(z_{0},z_{i_{\tau }}\right)\right)}^{1+{\bar{\rho }}_{i_{\tau }}}\right)}^{\frac{1}{1+{\bar{\rho }}_{i_{\tau }}}}\\ \; & \;{\left(\sum_{z_{0}\in Z_{0}}f_{0}\left(z_{0}\right){\left(\sum_{z_{j_{\tau }}\in Z_{j_{\tau }}}f_{j_{\tau }}^{\frac{1}{1+{\bar{\rho }}_{j_{\tau }}}}\left(z_{0},z_{j_{\tau }}\right)\right)}^{1+{\bar{\rho }}_{j_{\tau }}}\right)}^{\frac{{\bar{\rho }}_{i_{\tau }}}{1+{\bar{\rho }}_{i_{\tau }}}}. \end{array}$$

Next, putting Equation (A17) back in Equation (A10) and using the following inequality, proved in Appendix A.1, for $A_{i}\geq 0$ and $0\leq s_{i}\leq 1$

(A18) $$\begin{array}{c} \sum_{i,j\in K}A_{i}^{s_{i}}A_{j}^{1-s_{i}}\leq 2\left|K\right|\sum_{i\in K}A_{i} \end{array}$$

in the double summation over $i_{\tau }$ and $j_{\tau }$ in Equation (A10), the following upper bound holds

(A19) $$\begin{array}{c} \sum_{i_{\tau }\in K_{\tau }}\sum_{j_{\tau }\in K_{\tau }}\min_{s\geq 0}\min_{\rho \in [0,1]}{\bar{P}}_{e,i_{\tau^{c}}i_{\tau }}^{\tau ,j_{\tau }}\leq 2K_{\tau }\sum_{i_{\tau }\in K_{\tau }}\min_{\rho \in [0,1]}{\bar{P}}_{e,i_{\tau^{c}}i_{\tau }}^{\tau }, \end{array}$$

where we have moved the optimization over ${\bar{\rho }}_{\ell_{\tau }}$ inside the summation over $i_{\tau }$ and renamed ${\bar{\rho }}_{\ell_{\tau }}$ as ρ, with the dependence on the index $i_{\tau }$ kept implicit. Moreover, the expression for ${\bar{P}}_{e,i_{\tau^{c}}i_{\tau }}^{\tau }$ is in fact given by ${\bar{P}}_{e,i_{\tau^{c}}i_{\tau }}^{\tau ,j_{\tau }}$ in Equation (A11) after setting $i_{\tau }=j_{\tau }$, $s=\frac{1}{1+\rho }$ and rearranging terms, that is,

(A20) $$\begin{array}{cc} \;{\bar{P}}_{e,i_{\tau^{c}}i_{\tau }}^{\tau }=\sum_{\begin{array}{c} u_{\tau^{c}}\in A_{\tau^{c}}^{i_{\tau^{c}}}\\ x_{\tau^{c}}\in D_{\tau^{c},u_{\tau^{c}}}^{i_{\tau^{c}}} \end{array}}\; & P_{\tau^{c}}\left(u_{\tau^{c}}\right)Q_{\tau^{c}}^{i_{\tau^{c}}}\left(x_{\tau^{c}}|u_{\tau^{c}}\right)\sum_{y\in Y^{n}}{\left(\sum_{\begin{array}{c} u_{\tau }\in A_{\tau }^{i_{\tau }}\\ x_{\tau }\in D_{\tau ,u_{\tau }}^{i_{\tau }} \end{array}}P_{\tau |\tau^{c}}{\left(u_{\tau }|u_{\tau^{c}}\right)}^{\frac{1}{1+\rho }}Q_{\tau }^{i_{\tau }}\left(x_{\tau }|u_{\tau }\right)W{\left(y|x_{N}\right)}^{\frac{1}{1+\rho }}\right)}^{1+\rho }. \end{array}$$

It remains to factorize Equation (A20) into a product of symbol distributions in order to obtain a single-letter expression for the exponent. We start by upper bounding the summations over the input messages $u_{\tau^{c}}$ and $u_{\tau }$. For a list of users σ with corresponding messages $u_{\sigma }$, list of class indices $i_{\sigma }$ and some function $p_{\sigma }^{i_{\sigma }}\left(u_{\sigma }\right)$, we have that

(A21) $$\begin{array}{cc} \sum_{\begin{array}{c} u_{\sigma }\in A_{\sigma }^{i_{\sigma }} \end{array}}p_{\sigma }^{i_{\sigma }}\left(u_{\sigma }\right) & =\sum_{u_{\sigma }\in U_{\sigma }^{n}}p_{\sigma }^{i_{\sigma }}\left(u_{\sigma }\right)1\left\{u_{\sigma }\in A_{\sigma }^{i_{\sigma }}\right\}, \end{array}$$

where we used the definition of the message sets $A_{\sigma }^{i_{\sigma }}$ in Equation (12) and the identity

(A22) $$\begin{array}{c} \sum_{i\in K}f_{i}=\sum_{i\in N}f_{i}\;1\left\{i\in K\right\}. \end{array}$$

Using the upper bound

(A23) $$\begin{array}{c} 1\{a<b\leq c\}\leq \min_{\lambda^{L},\lambda^{U}\geq 0}{\left(\frac{b}{a}\right)}^{\lambda^{L}}{\left(\frac{c}{b}\right)}^{\lambda^{U}} \end{array}$$

for a,b,c>0 with $\lambda^{L},\lambda^{U}\geq 0$, together with the fact that the source-message classes are defined separately for each user to express the source message probabilities in terms of $P_{\sigma }^{\mathrm{ind}}\left(u_{\sigma }\right)=\prod_{\nu \in \sigma }P_{\nu }\left(u_{\nu }\right)$ similarly to Equation (2), we upper bound the r. h. s. of Equation (A21) as

(A24) $$\begin{array}{c} \sum_{\begin{array}{c} u_{\sigma }\in A_{\sigma }^{i_{\sigma }} \end{array}}p_{\sigma }^{i_{\sigma }}\left(u_{\sigma }\right)\leq \min_{\lambda_{\sigma }^{L,U}\geq 0}\sum_{u_{\sigma }\in U_{\sigma }^{n}}p_{\sigma }^{i_{\sigma }}\left(u_{\sigma }\right){\left(\frac{P_{\sigma }^{\mathrm{ind}}\left(u_{\sigma }\right)}{\gamma_{\sigma ,i_{\sigma }}^{n}}\right)}^{\lambda_{\sigma }^{L}}{\left(\frac{\gamma_{\sigma ,i_{\sigma }-1}^{n}}{P_{\sigma }^{\mathrm{ind}}\left(u_{\sigma }\right)}\right)}^{\lambda_{\sigma }^{U}}, \end{array}$$

where we jointly wrote $\lambda_{\sigma }^{L}$ and $\lambda_{\sigma }^{U}$ as $\lambda_{\sigma }^{L,U}$. Definining

(A25) $$\begin{array}{c} \Lambda_{\sigma }^{i_{\sigma }}\left(u_{\sigma }\right)={\left(\frac{P_{\sigma }^{\mathrm{ind}}\left(u_{\sigma }\right)}{\gamma_{\sigma ,i_{\sigma }}}\right)}^{\lambda_{\sigma }^{L}}{\left(\frac{\gamma_{\sigma ,i_{\sigma }-1}}{P_{\sigma }^{\mathrm{ind}}\left(u_{\sigma }\right)}\right)}^{\lambda_{\sigma }^{U}}. \end{array}$$

and taking into account that the sources are memoryless, we obtain that the summations w. r. t. the source messages $u_{\tau }$ and $u_{\tau^{c}}$ in Equation (A20) are upper bounded as

(A26) $$\begin{array}{c} \sum_{\begin{array}{c} u_{\sigma }\in A_{\sigma }^{i_{\sigma }} \end{array}}p_{\sigma }^{i_{\sigma }i_{\sigma }}\left(u_{\sigma }\right)\leq \min_{\lambda_{\sigma }^{L,U}}\sum_{u_{\sigma }\in U_{\sigma }^{n}}p_{\sigma }^{i_{\sigma^{c}}i_{\sigma }}\left(u_{\sigma }\right)\prod_{t=1}^{n}\Lambda_{\sigma }^{i_{\sigma }}\left(u_{\sigma ,t}\right). \end{array}$$

respecively for σ=τ and $\sigma =\tau^{c}$.

We proceed in a similar manner for the summations w. r. t. the codewords $x_{\tau }$ and $x_{\tau^{c}}$ in Equation (A20). For a list of users σ and some function $q_{\sigma }^{i_{\sigma }}\left(u_{\sigma },x_{\sigma }\right)$ implicitly defined, the summation over channel codewords $x_{\sigma }\in D_{\sigma ,u_{\sigma }}^{i_{\sigma }}$ can be upper bounded as:

(A27) $$\begin{array}{cc} \sum_{x_{\sigma }\in D_{\sigma ,u_{\sigma }}^{i_{\sigma }}}q_{\sigma }^{i_{\sigma }}\left(u_{\sigma },x_{\sigma }\right) & =\sum_{x_{\sigma }\in X_{\sigma }^{n}}q_{\sigma }^{i_{\sigma }}\left(u_{\sigma },x_{\sigma }\right)1\left\{x_{\sigma }\in D_{\sigma ,u_{\sigma }}^{i_{\sigma }}\right\} \end{array}$$

(A28) $$\begin{array}{cc} \; & =\sum_{x_{\sigma }\in X_{\sigma }^{n}}q_{\sigma }^{i_{\sigma }}\left(u_{\sigma },x_{\sigma }\right)\prod_{\nu \in \sigma }\prod_{\upsilon_{\nu }\in U_{\nu }}\prod_{\ell_{\nu }\in L_{\nu }}1\left\{\left|a_{\nu ,\upsilon_{\nu }}^{i_{\nu },\ell_{\nu }}\left(x_{\nu }\left(I_{\upsilon_{\nu }}\left(u_{\nu }\right)\right)\right)\right|\leq \delta_{\nu }\right\} \end{array}$$

(A29) $$\begin{array}{cc} \; & \leq \min_{r_{\sigma \upsilon_{\sigma }}^{\ell_{\sigma }},{\bar{r}}_{\sigma \upsilon_{\sigma }}^{\ell_{\sigma }}}\sum_{x_{\sigma }\in X_{\sigma }^{n}}q_{\sigma }^{i_{\sigma }}\left(u_{\sigma },x_{\sigma }\right)\prod_{\upsilon_{\sigma }\in U_{\sigma }}\prod_{\ell_{\sigma }\in L_{\sigma }}e^{r_{\sigma \upsilon_{\sigma }}^{\ell_{\sigma }}\;a_{\sigma ,\upsilon_{\sigma }}^{i_{\sigma },\ell_{\sigma }}\left(x_{\sigma }\left(I_{\upsilon_{\sigma }}\left(u_{\sigma }\right)\right)\right)+{\bar{r}}_{\sigma \upsilon_{\sigma }}^{\ell_{\sigma }}\;\delta_{\sigma }}, \end{array}$$

where we used Equation (A22) in Equation (A27), the fact that the codeword ensembles are defined separately for each user together with the definition of the ensemble cost constraints in Equation (23) and subcodewords $x_{\nu }\left(I_{u_{\nu }}\left(u_{\nu }\right)\right)$ in Equation (A28), and a variant of Equation (A23) proved in Appendix A.2,

(A30) $$\begin{array}{c} 1\left\{|a|\leq \delta \right\}\leq \min_{r,\bar{r}}e^{ra+\bar{r}\delta } \end{array}$$

for r∈R and $\bar{r}\geq 0$, in Equation (A29) for each indicator function of Equation (A28) and combined the product of exponentials over σ as a single exponential using the list notation. We continue by rewriting the double product over $\upsilon_{\sigma }$ and $\ell_{\sigma }$ in Equation (A29) as follows

(A31) $$\begin{array}{cc} \prod_{\upsilon_{\sigma }\in U_{\sigma }}\prod_{\ell_{\sigma }\in L_{\sigma }}e^{r_{\sigma \upsilon_{\sigma }}^{\ell_{\sigma }}\;a_{\sigma ,\upsilon_{\sigma }}^{i_{\sigma },\ell_{\sigma }}\left(x_{\sigma }\left(I_{\upsilon_{\sigma }}\left(u_{\sigma }\right)\right)\right)+{\bar{r}}_{\sigma \upsilon_{\sigma }}^{\ell_{\sigma }}\;\delta_{\sigma }} & =\prod_{\upsilon_{\sigma }\in U_{\sigma }}\prod_{\ell_{\sigma }\in L_{\sigma }}e^{{\bar{r}}_{\sigma \upsilon_{\sigma }}^{\ell_{\sigma }}\;\delta_{\sigma }}\;\;\;\prod_{t\in I_{\upsilon_{\sigma }}\left(u_{\sigma }\right)}e^{r_{\sigma \upsilon_{\sigma }}^{\ell_{\sigma }}\;a_{\sigma ,\upsilon_{\sigma }}^{i_{\sigma },\ell_{\sigma }}\left(x_{\sigma ,t}\left(I_{\upsilon_{\sigma }}\left(u_{\sigma }\right)\right)\right)} \end{array}$$

(A32) $$\begin{array}{cc} \; & =\beta_{\sigma }\prod_{t=1}^{n}R_{\sigma ,u_{\tau ,t}}^{i_{\tau }}\left(x_{\tau ,t}\right), \end{array}$$

where in Equation (A31) we wrote the cost function in terms of the symbol costs and in Equation (A32) we rearranged terms and introduced a factor $\beta_{\sigma }$ that depends on the list $\left\{{\bar{r}}_{\sigma \upsilon_{\sigma }}^{\ell_{\sigma }}\right\}$ and a function $R_{\sigma ,u_{\sigma }}^{i_{\sigma }}\left(x_{\sigma }\right)$ that depends on the list $\left\{r_{\sigma \upsilon_{\sigma }}^{\ell_{\sigma }}\right\}$ and are respectively given by

(A33) $$\begin{array}{c} \beta_{\sigma }=\prod_{\upsilon_{\sigma }\in U_{\sigma }}\prod_{\ell_{\sigma }\in L_{\sigma }}e^{{\bar{r}}_{\sigma \upsilon_{\sigma }}^{\ell_{\sigma }}\;\delta_{\sigma }}, \end{array}$$

(A34) $$\begin{array}{c} R_{\sigma ,u_{\sigma }}^{i_{\sigma }}\left(x_{\sigma }\right)=\prod_{\ell_{\sigma }\in L_{\sigma }}e^{r_{\sigma u_{\sigma }}^{\ell_{\sigma }}\;a_{\sigma ,u_{\sigma }}^{i_{\sigma },\ell_{\sigma }}\left(x_{\sigma }\right)}. \end{array}$$

Replacing Equation (A32) back into Equation (A29), we obtain that the summations over the codewords are upper bounded as

(A35) $$\begin{array}{cc} \sum_{x_{\sigma }\in D_{\sigma ,u_{\sigma }}^{i_{\sigma }}}q_{\sigma }^{i_{\sigma }}\left(u_{\sigma },x_{\sigma }\right) & \leq \min_{r_{\sigma u_{\sigma }}^{\ell_{\sigma }},{\bar{r}}_{\sigma u_{\sigma }}^{\ell_{\sigma }}}\sum_{x_{\sigma }\in X_{\sigma }^{n}}q_{\sigma }^{i_{\sigma }}\left(u_{\sigma },x_{\sigma }\right)\beta_{\sigma }\prod_{t=1}^{n}R_{\sigma ,u_{\sigma ,t}}^{i_{\sigma }}\left(x_{\tau ,t}\right). \end{array}$$

for both user lists σ=τ and $\sigma =\tau^{c}$.

We now combine Equations (A26) and (A35) for σ=τ to bound the summation inside the parenthesis in Equation (A20) as

(A36) $$\begin{array}{cc} \; & \sum_{\begin{array}{c} u_{\tau }\in A_{\tau }^{i_{\tau }}\\ x_{\tau }\in D_{\tau ,u_{\tau }}^{i_{\tau }} \end{array}}P_{\tau |\tau^{c}}{\left(u_{\tau }|u_{\tau^{c}}\right)}^{\frac{1}{1+\rho }}Q_{\tau }^{i_{\tau }}\left(x_{\tau }|u_{\tau }\right)W{\left(y|x_{N}\right)}^{\frac{1}{1+\rho }}\leq \\ \; & \;\leq \sum_{\begin{array}{c} u_{\tau }\in U_{\tau }^{n}\\ x_{\tau }\in X_{\tau }^{n} \end{array}}P_{\tau |\tau^{c}}{\left(u_{\tau }|u_{\tau^{c}}\right)}^{\frac{1}{1+\rho }}\min_{\begin{array}{c} \lambda_{\tau }^{L,U}\\ r_{\tau u_{\tau }}^{\ell_{\tau }},{\bar{r}}_{\tau u_{\tau }}^{\ell_{\tau }} \end{array}}\frac{\beta_{\tau }}{\Xi_{\tau }}\prod_{t=1}^{n}\Lambda_{\tau }^{i_{\tau }}\left(u_{\tau ,t}\right)Q_{\tau ,u_{\tau ,t}}^{i_{\tau }}\left(x_{\tau ,t}\right)R_{\tau ,u_{\tau ,t}}^{i_{\tau }}\left(x_{\tau ,t}\right)W{\left(y|x_{N}\right)}^{\frac{1}{1+\rho }}, \end{array}$$

where we expressed the distribution $Q_{\tau }^{i_{\tau }}\left(x_{\tau },u_{\tau }\right)$ in terms of the symbol-wise iid distribution $Q_{\tau ,u_{\tau }}^{i_{\tau }}\left(x\right)$ as in Equation (25). Since both source and channel are memoryless, we may now factorize and rearrange the expression in Equation (A36) into single-letter, symbolwise factors as

(A37) $$\begin{array}{c} \sum_{\begin{array}{c} u_{\tau }\in A_{\tau }^{i_{\tau }}\\ x_{\tau }\in D_{\tau ,u_{\tau }}^{i_{\tau }} \end{array}}P_{\tau |\tau^{c}}{\left(u_{\tau }|u_{\tau^{c}}\right)}^{\frac{1}{1+\rho }}Q_{\tau }^{i_{\tau }}\left(x_{\tau }|u_{\tau }\right)W{\left(y|x_{N}\right)}^{\frac{1}{1+\rho }}\leq \min_{\begin{array}{c} \lambda_{\tau }^{L,U}\\ r_{\tau u_{\tau }}^{\ell_{\tau }},{\bar{r}}_{\tau u_{\tau }}^{\ell_{\tau }} \end{array}}\frac{\beta_{\tau }}{\Xi_{\tau }}\prod_{t=1}^{n}g_{\tau }^{i_{\tau }}\left(u_{\tau^{c},t},x_{\tau^{c},t},y_{t}\right), \end{array}$$

where, for a list of users σ, the function $g_{\sigma }^{i_{\sigma }}\left(u_{\sigma^{c}},x_{\sigma^{c}},y\right)$ is defined as

(A38) $$\begin{array}{c} g_{\sigma }^{i_{\sigma }}\left(u_{\sigma^{c}},x_{\sigma^{c}},y\right)=\sum_{\begin{array}{c} u_{\sigma }\in U_{\sigma }\\ x_{\sigma }\in X_{\sigma } \end{array}}P_{\sigma |\sigma^{c}}{\left(u_{\sigma }|u_{\sigma^{c}}\right)}^{\frac{1}{1+\rho }}\Lambda_{\sigma }^{i_{\sigma }}\left(u_{\sigma }\right)Q_{\sigma ,u_{\sigma }}^{i_{\sigma }}\left(x_{\sigma }\right)R_{\sigma ,u_{\sigma }}^{i_{\sigma }}\left(x_{\sigma }\right)W{\left(y|x_{N}\right)}^{\frac{1}{1+\rho }}. \end{array}$$

Although not explicitly, the function $g_{\tau }^{i_{\tau }}\left(u_{\tau^{c}},x_{\tau^{c}},y\right)$ in Equation (A37) depends on several optimization parameters, namely $\rho ,\lambda_{\tau }^{L,U},r_{\tau u_{\tau }}^{\ell_{\tau }},{\bar{r}}_{\tau u_{\tau }}^{\ell_{\tau }}$, which depend in turn on the error-event type τ and class indices $i_{\tau }$ and $i_{\tau^{c}}$.

Again, we use Equations (A26) and (A35) for $\sigma =\tau^{c}$ and the fact that the source is memoryless to upper bound the summation outside the parenthesis in Equation (A20) as

(A39) $$\begin{array}{c} \sum_{\begin{array}{c} u_{\tau^{c}}\in A_{\tau^{c}}^{i_{\tau^{c}}}\\ x_{\tau^{c}}\in D_{\tau^{c},u_{\tau^{c}}}^{i_{\tau^{c}}} \end{array}}P_{\tau^{c}}\left(u_{\tau^{c}}\right)Q_{\tau^{c}}^{i_{\tau^{c}}}\left(x_{\tau^{c}}|u_{\tau^{c}}\right)\leq \;\\ \leq \min_{\begin{array}{c} \lambda_{\tau^{c}}^{L,U}\\ r_{\tau^{c}u_{\tau^{c}}}^{\ell_{\tau^{c}}},{\bar{r}}_{\tau^{c}u_{\tau^{c}}}^{\ell_{\tau^{c}}} \end{array}}\sum_{\begin{array}{c} u_{\tau^{c}}\in U_{\tau^{c}}^{n}\\ x_{\tau^{c}}\in X_{\tau^{c}}^{n} \end{array}}\frac{\beta_{\tau^{c}}}{\Xi_{\tau^{c}}}\prod_{t=1}^{n}P_{\tau^{c}}\left(u_{\tau^{c},t}\right)\Lambda_{\tau^{c}}^{i_{\tau^{c}}}\left(u_{\tau^{c},t}\right)Q_{\tau^{c},u_{\tau^{c},t}}^{i_{\tau^{c}}}\left(x_{\tau^{c},t}\right)R_{\tau^{c},u_{\tau^{c},t}}^{i_{\tau^{c}}}\left(x_{\tau^{c},t}\right). \end{array}$$

Substituting Equations (A37) and (A39) in Equation (A20), the resulting expression back into Equation (A19) and then into Equation (A10), we get

(A40) $$\begin{array}{c} \;{\bar{P}}_{e}^{\tau }\leq 2K_{\tau }\sum_{\begin{array}{c} i_{\tau^{c}}\in K_{\tau^{c}}\\ i_{\tau }\in K_{\tau } \end{array}}\min_{\begin{array}{c} \rho ,\lambda_{\tau^{c}}^{L,U}\\ r_{\tau^{c}u_{\tau^{c}}}^{\ell_{\tau^{c}}},{\bar{r}}_{\tau^{c}u_{\tau^{c}}}^{\ell_{\tau^{c}}} \end{array}}\sum_{\begin{array}{c} u_{\tau^{c}}\in U_{\tau^{c}}^{n}\\ x_{\tau^{c}}\in X_{\tau^{c}}^{n} \end{array}}\frac{\beta_{\tau^{c}}}{\Xi_{\tau^{c}}}\left(\prod_{t=1}^{n}P_{\tau^{c}}\left(u_{\tau^{c},t}\right)\Lambda_{\tau^{c}}^{i_{\tau^{c}}}\left(u_{\tau^{c},t}\right)Q_{\tau^{c},u_{\tau^{c},t}}^{i_{\tau^{c}}}\left(x_{\tau^{c},t}\right)R_{\tau^{c},u_{\tau^{c},t}}^{i_{\tau^{c}}}\left(x_{\tau^{c},t}\right)\right)\;\\ \left(\;\sum_{y\in Y^{n}}{\left(\frac{\beta_{\tau }}{\Xi_{\tau }}\right)}^{1+\rho }\;\;\;\;\min_{\begin{array}{c} \lambda_{\tau }^{L,U}\\ r_{\tau u_{\tau }}^{\ell_{\tau }},{\bar{r}}_{\tau u_{\tau }}^{\ell_{\tau }} \end{array}}\prod_{t=1}^{n}g_{\tau }^{i_{\tau }}{\left(u_{\tau^{c},t},x_{\tau^{c},t},y_{t}\right)}^{1+\rho }\right). \end{array}$$

Let us define now the function $h_{\sigma }^{i_{\sigma },i_{\sigma^{c}}}$ of the user set σ and the class indices $i_{\sigma }$ and $i_{\sigma^{c}}$ as

(A41) $$\begin{array}{c} h_{\sigma }^{i_{\sigma },i_{\sigma^{c}}}=\sum_{\begin{array}{c} u_{\sigma }\in U_{\sigma },x_{\sigma }\in X_{\sigma },y\in Y \end{array}}P_{\sigma }\left(u_{\sigma }\right)\Lambda_{\sigma }^{i_{\sigma }}\left(u_{\sigma }\right)Q_{\sigma ,u_{\sigma }}^{i_{\sigma }}\left(x_{\sigma }\right)R_{\sigma ,u_{\sigma }}^{i_{\sigma }}\left(x_{\sigma }\right)\;g_{\sigma^{c}}^{i_{\sigma^{c}}}{\left(u_{\sigma },x_{\sigma },y\right)}^{1+\rho }. \end{array}$$

With this definition, we can rewrite Equation (A40) in a compact manner as

(A42) $$\begin{array}{cc} {\bar{P}}_{e}^{\tau } & \leq 2K_{\tau }\sum_{\begin{array}{c} i_{N}\in K_{N} \end{array}}\min_{\begin{array}{c} \rho ,\lambda_{N}^{L,U},r_{Nu_{N}}^{\ell_{N}},{\bar{r}}_{Nu_{N}}^{\ell_{N}} \end{array}}\frac{\beta_{\tau^{c}}}{\Xi_{\tau^{c}}}{\left(\frac{\beta_{\tau }}{\Xi_{\tau }}\right)}^{1+\rho }\prod_{t=1}^{n}h_{\tau^{c}}^{i_{\tau^{c}},i_{\tau }}, \end{array}$$

where we have also combined the complementary sets $i_{\tau^{c}}$ and $i_{\tau }$ into $i_{N}$, and similarly for $\lambda_{N}^{L}$, $\lambda_{N}^{U}$, $r_{Nu_{N}}^{\ell_{N}}$, and ${\bar{r}}_{Nu_{N}}^{\ell_{N}}$. Finally, substituting Equation (A42) into Equation (A10) and then back into Equation (A2), taking (minus) the logarithm of the bound on ${\bar{P}}_{e}$, dividing the result by *n*, and the limit as n→∞, we obtain a lower bound $E_{\mathrm{KL}}^{\mathrm{cost}}$ to the exponent of the generalized cost-constrained ensemble $E_{\mathrm{KL}}^{\mathrm{cost}}$, namely

(A43) $$\begin{array}{cc} E_{K}^{\mathrm{cost}}L & =\min_{\tau ,i_{N}}\max_{\begin{array}{c} \rho ,\lambda_{N}^{L,U},r_{Nu_{N}}^{\ell_{N}} \end{array}}\left\{-\log h_{\tau^{c}}^{i_{\tau^{c}},i_{\tau }}\right\}, \end{array}$$

where we have used that as n→∞, the quantities $2K_{\tau }$, $\frac{\beta_{\tau^{c}}}{\Xi_{\tau^{c}}}$, and ${\left(\frac{\beta_{\tau }}{\Xi_{\tau }}\right)}^{1+\rho }$ are subexponential in the blocklength *n* and do not contribute to the exponent, accordingly removed ${\bar{r}}_{\tau^{c}u_{\tau^{c}}}^{\ell_{\tau^{c}}}$ and ${\bar{r}}_{\tau u_{\tau }}^{\ell_{\tau }}$ from the optimization parameter list, and finally used that the exponential decay of the error probability in Equation (A2) will be dominated by the worst error type τ and the worst classes assignment $i_{N}$. It will prove convenient to the express the exponent in terms of a Gallager function $E_{\tau }^{i_{N}}\left(\rho ,\lambda_{N}^{L,U},r_{Nu_{N}}^{\ell_{N}}\right)$, defined as

(A44) $$\begin{array}{c} E_{\tau }^{i_{N}}\left(\rho ,\lambda_{N}^{L,U},r_{Nu_{N}}^{\ell_{N}}\right)=-\log h_{\tau^{c}}^{i_{\tau^{c}},i_{\tau }}. \end{array}$$

Substituted the expression for $h_{\tau^{c}}^{i_{\tau^{c}},i_{\tau }}$ in Equation (A40), where $\Lambda_{\tau }^{i_{\tau }}\left(u_{\tau }\right)$ and $R_{\nu ,u_{\nu }}^{i_{\nu }}\left(x_{\nu }\right)$ are respectively given by Equations (A25) and (A34), we may express $E_{\tau }^{i_{N}}\left(\rho ,\lambda_{N}^{L,U},r_{Nu_{N}}^{\ell_{N}}\right)$ as

(A45) EτiN(ρ,λNL,U,rNuNℓN)=−log∑uτc,xτc,yPτc(uτc)Λτciτc(uτc)Qτc,uτciτc(xτc)Rτc,uτciτc(xτc)=−log(∑uτ,xτPτ|τc(uτ|uτc)11+ρΛτiτ(uτ)Qτ,uτiτ(xτ)Rτ,uτiτ(xτ)W(y|xN)11+ρ1+ρ,

or equivalently in the alternative form

(A46) $$\begin{array}{cc} \; & \;E_{\tau }^{i_{N}}\left(\rho ,\lambda_{N}^{L,U},r_{Nu_{N}}^{\ell_{N}}\right)=\\ \; & \;-\log\sum_{u_{\tau^{c}},x_{\tau^{c}},y}(\sum_{u_{\tau },x_{\tau }}P_{N}{\left(u_{N}\right)}^{\frac{1}{1+\rho }}\Lambda_{N}^{i_{N}}\left(u_{N}\right)Q_{\tau ,u_{\tau }}^{i_{\tau }}\left(x_{\tau }\right)R_{N,u_{N}}^{i_{N}}\left(x_{N}\right){\left(Q_{\tau^{c},u_{\tau^{c}}}^{i_{\tau^{c}}}\left(x_{\tau^{c}}\right)W{\left(y|x_{N}\right)}^{\frac{1}{1+\rho }}\right)}^{1+\rho }, \end{array}$$

where in Equation (A46) we have moved the product

$$P_{\tau^{c}}\left(u_{\tau^{c}}\right)\Lambda_{\tau^{c}}^{i_{\tau^{c}}}\left(u_{\tau^{c}}\right)Q_{\tau^{c},u_{\tau^{c}}}^{i_{\tau^{c}}}\left(x_{\tau^{c}}\right)R_{\tau^{c},u_{\tau^{c}}}^{i_{\tau^{c}}}\left(x_{\tau^{c}}\right)$$

inside the parenthesis and merged terms in τ and $\tau^{c}$ as done above, as well as redefined the optimization parameters $\frac{\lambda_{\tau^{c}}^{L}}{1+\rho }$, $\frac{\lambda_{\tau^{c}}^{L}}{1+\rho }$, and $\frac{r_{\tau^{c}u_{\tau^{c}}}^{\ell_{\tau^{c}}}}{1+\rho }$ as $\lambda_{\tau^{c}}^{L}$, $\lambda_{\tau^{c}}^{U}$, and $r_{\tau^{c}u_{\tau^{c}}}^{\ell_{\tau^{c}}}$ respectively.

### Appendix A.1. Proof of Equation (A18)

A sketch of the proof of the inequality in Equation (A18) proceeds as follows:

(A47) $$\begin{array}{cc} \sum_{i,j\in K}A_{i}^{s_{i}}A_{j}^{1-s_{i}}\; & \leq \sum_{i,j\in K}\left(s_{i}A_{i}+\left(1-s_{i}\right)A_{j}\right) \end{array}$$

(A48) $$\begin{array}{cc} \; & \leq \sum_{i,j\in K}\left(A_{i}+A_{j}\right) \end{array}$$

(A49) $$\begin{array}{cc} \; & =2|K|\sum_{i\in K}A_{i} \end{array}$$

where Equation (A47) follows from the inequality between arithmetic and geometric means and in Equation (A48) we used that 0≤s≤1.

### Appendix A.2. Proof of Equation (A30)

We have the following

(A50) $$\begin{array}{cc} 1\{|a|\leq \delta \} & =1\{-\delta \leq a\}1\{a\leq \delta \} \end{array}$$

(A51) $$\begin{array}{cc} \; & =1\left\{e^{-\delta }\leq e^{a}\right\}1\left\{e^{a}\leq e^{+\delta }\right\} \end{array}$$

(A52) $$\begin{array}{cc} \; & \leq e^{r_{-}\left(a+\delta \right)}e^{r_{+}\left(\delta -a\right)} \end{array}$$

(A53) $$\begin{array}{cc} \; & =e^{ra+\bar{r}\delta } \end{array}$$

for $r_{-},r_{+}\geq 0$ or equivalently $r=r_{-}-r_{+}\in R$ and $\bar{r}=r_{-}+r_{+}\geq 0$. The bound in Equation (A53) can be optimized w. r. t. *r* and $\hat{r}$.

## Appendix B. Computation of the Optimum Multi-Class Thresholds

In this section we find some conditions describing the optimum partitioning of the source-message set into classes for the optimization of the exponent in Equation (30). For simplicity, let each user ν∈N have two classes, $K_{\nu }=2$.

From the class definition in Equation (12) with $K_{\nu }=2$, we have that $\gamma_{\nu ,2}=0$ and $\gamma_{\nu ,0}=1$, so we need find just one optimum $\gamma_{\nu ,1}$ for each user, which redefine as $\gamma_{\nu }$. Optimizing the exponent in Equation (30) over $\gamma_{N}$ gives

(A54) maxrNuNℓN0≤γN≤1Ecost=maxrNuNℓN0≤γN≤1minrNuNℓNiNminrNuNℓNτmaxρ,λNL,U,rNuNℓNEτiNρ,λNL,U,rNuNℓN,

where one of the parameters $\lambda_{N}^{L}$ or $\lambda_{N}^{U}$ is zero for each $i_{N}$, as the corresponding constraint is absent. For each $\gamma_{N}$, we have a minimization over $2^{N}$ assignments $i_{N}$. Following the same steps as in refs. [31] (Sec. 4.1.2) and [31] (Lemma 4.3), we find that $E_{\tau }^{i_{N}}\left(\gamma_{N}\right)$ defined, with some abuse of notation, as

(A55) $$\begin{array}{c} E_{\tau }^{i_{N}}\left(\gamma_{N}\right)=\max_{\begin{array}{c} \rho ,\lambda_{N}^{L,U},r_{Nu_{N}}^{\ell_{N}} \end{array}}E_{\tau }^{i_{N}}\left(\rho ,\lambda_{N}^{L,U},r_{Nu_{N}}^{\ell_{N}}\right), \end{array}$$

is a non-decreasing (resp. non-increasing) function with respect to $\gamma_{\nu }$ for $i_{N}=\left[i_{\nu },i_{\nu^{c}}\right]$ with $i_{\nu }=1$ (resp. $i_{\nu }=2$), irrespective of the values of $i_{\nu^{c}}$ and of ν. For the sake of completeness, we present an independent proof of this fact here. Let $i_{\nu }=1$ and τ be arbitrary. Using Equation (31), the function $E_{\tau }^{i_{N}}\left(\gamma_{N},\rho \right)$ has the form $-\log\left(\sum_{z}f_{1}\left(z\right)\backslash \gamma_{\nu }^{\lambda_{\nu }^{L}}\right))$ for some function $f_{1}\left(z\right)$, as all $\gamma_{N}$ are independent from each other, regardless the value of $i_{\nu^{c}}$. Since $\lambda_{\nu }^{L}\geq 0$, the function $E_{\tau }^{i_{N}}\left(\gamma_{N},\rho \right)$ in Equation (A55) is non-decreasing with respect to $\gamma_{\nu }$. When $i_{\nu }=2$, this function $E_{\tau }^{i_{N}}\left(\gamma_{N},\rho \right)$ has the form $-\log\left(\sum_{z}f_{2}\left(z\right)\gamma_{\nu }^{\lambda_{\nu }^{U}}\right))$ for some $f_{2}\left(z\right)$, and is therefore non-increasing. This behavior will not change after taking maximization over ρ. As the minimum of monotonic functions is monotonic, the function $E_{\tau }^{i_{N}}\left(\gamma_{N}\right)$ is non-decreasing (non-increasing) with respect to $\gamma_{\nu }$, when $i_{\nu }=1$ ($i_{\nu }=2$).

For any ν and fixed $\gamma_{\nu^{c}}$, we may write the optimization problem in Equation (A54) as

(A56) $$\begin{array}{c} \max_{\gamma_{\nu^{c}}}\max_{\gamma_{\nu }}\min_{i_{\nu }}\min_{i_{\nu^{c}}}\min_{\tau }E_{\tau }^{\left[i_{\nu },i_{\nu^{c}}\right]}\left([\gamma_{\nu },\gamma_{\nu^{c}}]\right). \end{array}$$

The optimization problem $\max_{\gamma_{\nu }}\min_{i_{\nu }}\min_{i_{\nu^{c}}}\min_{\tau }E_{\tau }^{\left[i_{\nu },i_{\nu^{c}}\right]}\left([\gamma_{\nu },\gamma_{\nu^{c}}]\right)$ satisfies the following lemma, proved in Appendix B.1, with $\gamma =\gamma_{\nu }$, $i=i_{\nu }$, and $k_{i}\left(\gamma \right)=\min_{i_{\nu^{c}}}\min_{\tau }E_{\tau }^{\left[i_{\nu },i_{\nu^{c}}\right]}\left([\gamma_{\nu },\gamma_{\nu^{c}}]\right)$.

**Lemma** **A1.**
*Let $k_{1}\left(\gamma \right)$ and $k_{2}\left(\gamma \right)$ be respectively continuous non-decreasing and non-increasing functions with respect to γ∈[0,1]. The optimal $\gamma^{☆}$ maximizing $\min_{i=1,2}k_{i}\left(\gamma \right)$ satisfies the following equation*

(A57) $$\begin{array}{c} k_{1}\left(\gamma^{☆}\right)=k_{2}\left(\gamma^{☆}\right). \end{array}$$

*When Equation (A57) does not have any solution, we have $\gamma^{☆}=0$ if $k_{1}\left(0\right)>k_{2}\left(0\right)$, and $\gamma^{☆}=1$ otherwise.*

Therefore, the optimal $\gamma_{\nu }^{☆}$ satisfies

(A58) $$\begin{array}{c} \min_{i_{\nu^{c}}}\min_{\tau }E_{\tau }^{\left[1,i_{\nu^{c}}\right]}\left([\gamma_{\nu }^{*},\gamma_{\nu^{c}}]\right)=\min_{i_{\nu^{c}}}\min_{\tau }E_{\tau }^{\left[2,i_{\nu^{c}}\right]}\left([\gamma_{\nu }^{*},\gamma_{\nu^{c}}]\right), \end{array}$$

if Equation (A58) has a solution. If not, the inequality $\min_{i_{\nu^{c}}}\min_{\tau }E_{\tau }^{\left[1,i_{\nu^{c}}\right]}\left([0,\gamma_{\nu^{c}}]\right)>\min_{i_{\nu^{c}}}\min_{\tau }E_{\tau }^{\left[1,i_{\nu^{c}}\right]}\left([0,\gamma_{\nu^{c}}]\right)$ is satisfied, we have $\gamma_{\nu }^{☆}=0$ or $\gamma_{\nu }^{☆}=1$ otherwise. Since Equation (A58) holds for any ν, evaluating it for each ν gives a system of equations for the computation of the optimal thresholds.

In ref. [31] (Sec. 3.2.1.1), we give a graphical interpretation of the solutions to Equation (A58) and outline the relevant differences with the single-user case. We observe a strong coupling between the exponent and the thresholds that prevents to find the optimal number of classes, suggesting that, unlike the single-user case, two classes might not be sufficient.

### Proof of Lemma A1

The relative behaviour of a non-decreasing function with a non-increasing function can be categorized in three cases.

1. If $k_{1}\left(0\right)<k_{2}\left(0\right)$ and $k_{1}\left(1\right)>k_{2}\left(1\right)$, there exists a $\gamma^{☆}$ such that $k_{1}\left(\gamma^{☆}\right)=k_{2}\left(\gamma^{☆}\right)$. In this case, the function $\min_{i}k_{i}\left(\gamma \right)$ is non-decreasing from $\left[0,\gamma^{☆}\right)$, and non-increasing from $\left(\gamma^{☆},1\right]$. Thus, the maximum over γ of $\min_{i}k_{i}\left(\gamma \right)$ occurs at $\gamma =\gamma^{☆}$.
2. If $k_{1}\left(0\right)<k_{2}\left(0\right)$ and $k_{1}\left(1\right)<k_{2}\left(1\right)$, $k_{1}\left(\gamma \right)$ and $k_{2}\left(\gamma \right)$ do not cross in γ∈[0,1]. Hence, we have $\min_{i}k_{i}\left(\gamma \right)=k_{1}\left(\gamma \right)$ and obviously since it is an non-decreasing function the maximum over γ occurs at $\gamma =\gamma^{☆}=1$.
3. When $k_{1}\left(0\right)\geq k_{2}\left(0\right)$, we have $\min_{i}k_{i}\left(\gamma \right)=k_{2}\left(\gamma \right)$ and hence $\gamma^{☆}=0$.

## Appendix C. Properties of the Modified Gallager Source Function

In this appendix, we study the modified Gallager source function $E_{s,\tau }^{i_{N}}$ in Equation (45) involved in the achievable exponent for the md-iid ensemble. For the sake of simplicity, we consider the rather illustrative case of N=2 users, each having a $K_{\nu }=2$-class partition of the source messages with thresholds $i_{N}$ where N={1,2}. From the definition of the sets $A_{\nu }^{i_{\nu }}$ in Equation (12) with $\gamma_{\nu ,0}=1$, $\gamma_{\nu ,1}=\gamma_{\nu }$ and $\gamma_{\nu ,2}=0$, the two message sets

(A59) $$\begin{array}{c} A_{\nu }^{1}\left(\gamma_{\nu }\right)=\left\{u_{\nu }\in U_{\nu }^{n}\;:\;P_{\nu }\left(u_{\nu }\right)\geq \gamma_{\nu }^{n}\right\}, \end{array}$$

(A60) $$\begin{array}{c} A_{\nu }^{2}\left(\gamma_{\nu }\right)=\left\{u_{\nu }\in U_{\nu }^{n}\;:\;P_{\nu }\left(u_{\nu }\right)<\gamma_{\nu }^{n}\right\}, \end{array}$$

are specified using a single threshold $\gamma_{\nu }$ for each user ν∈{1,2}. With some abuse of notation, we include the optimization w. r. t. $\lambda_{N}^{L,U}$ and make explicit the dependence on the thresholds $\gamma_{N}$ in the expression of the source function $E_{s,\tau }^{i_{N}}$ in Equation (45), namely

(A61) $$E_{s,\tau }^{i_{N}}\left(\rho ,P_{N},\gamma_{N}\right)=\min_{\lambda_{N}^{L,U}\geq 0}\log\sum_{u_{\tau^{c}}}{\left(\sum_{u_{\tau }}P_{N}{\left(u_{N}\right)}^{\frac{1}{1+\rho }}\Lambda_{N}^{i_{N}}\left(u_{N}\right)\right)}^{1+\rho }.$$

For $i_{\nu }=1$, the set $A_{\nu }^{1}\left(\gamma_{\nu }\right)$ in Equation (A59) has no upper threshold, hence we find that the optimal parameter $\lambda_{\nu }^{U}$ in this case is ${\hat{\lambda }}_{\nu }^{U}=0$. Similarly for $i_{\nu }=2$, we obtain that ${\hat{\lambda }}_{\nu }^{L}=0$. As a consequence and without any loss of generality, we define $\lambda_{\nu }=\lambda_{\nu }^{L}$ for $i_{\nu }=1$, and $\lambda_{\nu }=\lambda_{\nu }^{U}$ for $i_{\nu }=2$, and further simplify Equation (A61) to the following optimization problem

(A62) $$\begin{array}{c} E_{s,\tau }^{i_{N}}\left(\rho ,P_{N},\gamma_{N}\right)=\min_{\lambda_{N}\geq 0}\log\sum_{u_{\tau^{c}}}{\left(\sum_{u_{\tau }}P_{N}{\left(u_{N}\right)}^{\frac{1}{1+\rho }}{\left(\frac{\gamma_{1}}{P_{1}\left(u_{1}\right)}\right)}^{{\left(-1\right)}^{i_{1}}\lambda_{1}}{\left(\frac{\gamma_{2}}{P_{2}\left(u_{2}\right)}\right)}^{{\left(-1\right)}^{i_{2}}\lambda_{2}}\right)}^{1+\rho }, \end{array}$$

where we also used the definition of the functions $\Lambda_{\sigma }^{i_{\sigma }}$ in Equation (32) with σ={1,2}. We recall that $P_{1}$ and $P_{2}$ are the marginal distributions for users ν=1 and ν=2, respectively, and the indices $i_{\nu }\in \left\{1,2\right\}$ indicate that user ν transmits a source message selected from the class $A_{\nu }^{i_{\nu }}\left(\gamma_{\nu }\right)$ in Equations (A59) and (A60). It can be shown that the objective function in the r. h. s. of Equation (A62) is convex w. r. t. both $\lambda_{1}$ and $\lambda_{2}$. Hence, the minimizers ${\hat{\lambda }}_{1}$ and ${\hat{\lambda }}_{2}$ in the source function $E_{s,\tau }^{i_{N}}\left(\rho ,P_{N},\gamma_{N}\right)$ are respectively given by ${\hat{\lambda }}_{1}=\max\left\{\lambda_{1}^{☆},0\right\}$ and ${\hat{\lambda }}_{2}=\max\left\{\lambda_{2}^{☆},0\right\}$, where $\lambda_{1}^{☆}$ and $\lambda_{2}^{☆}$ are the unique solution after setting the partial derivatives of the r. h. s. of Equation (A62) to zero. Two special cases can be obtained from Equation (A62).

The first case is when $\gamma_{\nu }=1$ for ν∈{1,2}, implying that no message partition happens whatsoever. In such a case, we have that ${\hat{\lambda }}_{1}={\hat{\lambda }}_{2}=0$ and Equation (A62) reduces to the joint source-channel coding source function for correlated-sources in Equation (34), i.e.,

(A63) $$\begin{array}{c} E_{s,\tau }\left(\rho ,P_{N}\right)=\log\sum_{u_{\tau^{c}}}{\left(\sum_{u_{\tau }}P_{N}{\left(u_{N}\right)}^{\frac{1}{1+\rho }}\right)}^{1+\rho }. \end{array}$$

The second one is the case of independent sources. Substituting $P_{N}=P_{1}P_{2}$ in Equation (A62), after some algebra, we obtain that $E_{s,\tau }^{i_{N}}\left(\rho ,P_{N},\gamma_{N}\right)$ can be split into two terms as

(A64) $$\begin{array}{c} E_{s,\tau }^{i_{N}}\left(\rho ,P_{N},\gamma_{N}\right)=E_{s}^{i_{\tau }}\left(\rho ,P_{\tau },\gamma_{\tau }\right)+E_{s}^{i_{\tau^{c}}}\left(0,P_{\tau^{c}},\gamma_{\tau^{c}}\right), \end{array}$$

where we defined the function $E_{s}^{i}\left(\rho ,P,\gamma \right)$ as

(A65) $$\begin{array}{c} E_{s}^{i}\left(\rho ,P,\gamma \right)=\min_{\lambda_{}\geq 0}\log{\left(\sum_{u}P{\left(u\right)}^{\frac{1}{1+\rho }}{\left(\frac{\gamma }{P(u)}\right)}^{{\left(-1\right)}^{i}\lambda }\right)}^{1+\rho } \end{array}$$

for arbitrary class index i∈{1,2}, source distribution *P* and threshold γ. First, we find that the unique solution after setting the derivative of the r. h. s. of Equation (A65) to zero, denoted as $\lambda^{☆}$, is implicitly given by

(A66) $$\begin{array}{c} \frac{\sum_{u}P{\left(u\right)}^{\frac{1}{1+\alpha^{☆}}}\log P\left(u\right)}{\sum_{u}P{\left(u\right)}^{\frac{1}{1+\alpha^{☆}}}}=\log\left(\gamma \right), \end{array}$$

where we made the convenient change of variable

(A67) $$\begin{array}{c} \frac{1}{1+\alpha^{☆}}=\frac{1}{1+\rho }-{\left(-1\right)}^{i}\lambda^{☆}. \end{array}$$

Although not made explicit, $\lambda^{☆}$ depends on the triplet (i,P,ρ,γ). When $\lambda^{☆}<0$, or equivalently when

(A68) $$\begin{array}{c} {\left(-1\right)}^{i}\left(\frac{1}{1+\rho }-\frac{1}{1+\alpha^{☆}}\right)<0 \end{array}$$

we have that $\hat{\lambda }=\max\left(0,\lambda^{☆}\right)=0$, implying that Equation (A65) simplifies to

(A69) $$\begin{array}{c} E_{s}^{i}\left(\rho ,P,\gamma \right)=E_{s}\left(\rho ,P\right), \end{array}$$

where $E_{s}\left(\rho ,P\right)$ is the Gallager source function

(A70) $$\begin{array}{c} E_{s}\left(\rho ,P\right)=\log{\left(\sum_{u}P{\left(u\right)}^{\frac{1}{1+\rho }}\right)}^{1+\rho }. \end{array}$$

Otherwise, when $\hat{\lambda }=\lambda^{☆}\geq 0$, a regime given by the following inequality

(A71) $$\begin{array}{c} {\left(-1\right)}^{i}\left(\frac{1}{1+\rho }-\frac{1}{1+\alpha^{☆}}\right)\geq 0 \end{array}$$

we may substitute $\lambda =\lambda^{☆}$ in the objective function in Equation (A65) to obtain

(A72) $$\begin{array}{c} E_{s}^{i}\left(\rho ,P,\gamma \right)=\left(1+\rho \right)\log\left(\sum_{u}P{\left(u\right)}^{\frac{1}{1+\alpha^{☆}}}\right)+\frac{\alpha^{☆}-\rho }{1+\alpha^{☆}}\log\left(\gamma \right), \end{array}$$

where we wrote the expression in terms of $\alpha^{☆}$. Using Equation (A66) into Equation (A72) to replace log(γ), we get

(A73) $$\begin{array}{c} E_{s}^{i}\left(\rho ,P,\gamma \right)=\left(1+\rho \right)\log\left(\sum_{u}P{\left(u\right)}^{\frac{1}{1+\alpha^{☆}}}\right)+\frac{\alpha^{☆}-\rho }{1+\alpha^{☆}}\frac{\sum_{u}P{\left(u\right)}^{\frac{1}{1+\alpha^{☆}}}\log P\left(u\right)}{\sum_{u}P{\left(u\right)}^{\frac{1}{1+\alpha^{☆}}}}. \end{array}$$

After some algebra, we are able to express the former equation in terms of the derivative of the $E_{s}$-function in Equation (A70), given by

(A74) $$\begin{array}{c} E_{s}^{\prime }\left(\rho ,P\right)=\log\left(\sum_{u}P{\left(u\right)}^{\frac{1}{1+\rho }}\right)-\frac{1}{1+\rho }\frac{\sum_{u}P{\left(u\right)}^{\frac{1}{1+\rho }}\log P\left(u\right)}{\sum_{u}P{\left(u\right)}^{\frac{1}{1+\rho }}}, \end{array}$$

and the $E_{s}$-function itself, as

(A75) $$\begin{array}{c} E_{s}^{i}\left(\rho ,P,\gamma \right)=E_{s}\left(\alpha^{☆},P\right)+\left(\rho -\alpha^{☆}\right)E_{s}^{\prime }\left(\alpha^{☆},P\right). \end{array}$$

We may finally combine Equations (A69) and (A75), with the respective ranges in Equations (A68) and (A71) to write the $E_{s}^{i}\left(\rho ,P,\gamma \right)$ function in Equation (A65) piecewise as

(A76) $$\begin{array}{c} E_{s}^{1}\left(\rho ,P,\gamma \right)=\left\{\begin{array}{cc} E_{s}\left(\rho ,P\right) & \frac{1}{1+\rho }\geq \frac{1}{1+\alpha^{☆}},\\ E_{s}\left(\alpha^{☆},P\right)+E_{s}^{\prime }\left(\alpha^{☆}\right)\left(\rho -\alpha^{☆}\right) & \frac{1}{1+\rho }<\frac{1}{1+\alpha^{☆}}, \end{array}\right. \end{array}$$

and

(A77) $$\begin{array}{c} E_{s}^{2}\left(\rho ,P,\gamma \right)=\left\{\begin{array}{cc} E_{s}\left(\rho ,P\right) & \frac{1}{1+\rho }<\frac{1}{1+\alpha^{☆}},\\ E_{s}\left(\alpha^{☆},P\right)+E_{s}^{\prime }\left(\alpha^{☆}\right)\left(\rho -\alpha^{☆}\right) & \frac{1}{1+\rho }\geq \frac{1}{1+\alpha^{☆}}. \end{array}\right. \end{array}$$

where $\alpha^{☆}$ is the solution to the implicit Equation (A66), hence recovering the source error exponent functions of the md-iid ensemle described in ref. [8] (Lemma 1). The source functions $E_{s}^{1}\left(\rho ,P,\gamma \right)$ and $E_{s}^{2}\left(\rho ,P,\gamma \right)$ follow the Gallager function in Equation (A70) for a certain interval of ρ, and are the straight-line tangent beyond that interval. The tangent point $\alpha^{☆}$ is a function of the distribution *P* and of the multi-class threshold γ.

Once $E_{s}^{i}\left(\rho ,P,\gamma \right)$ in Equation (A65) is fully characterized, we may now discuss the correlated-sources error function $E_{s,\tau }^{i_{N}}\left(\rho ,P_{N},\gamma_{N}\right)$ in Equation (A64) in terms of the error type τ. We start with the third error type τ={1,2}, for which since $\tau^{c}=\emptyset$, we have that

(A78) $$\begin{array}{c} E_{s,\tau }^{i_{N}}\left(\rho ,P_{N},\gamma_{N}\right)=E_{s}^{i_{1}}\left(\rho ,P_{1},\gamma_{1}\right)+E_{s}^{i_{2}}\left(\rho ,P_{2},\gamma_{2}\right), \end{array}$$

namely the superposition of two $E_{s}^{i}$ functions as the ones in Equations (A76) and (A77), one for each user. For the remaining of this appendix, we consider the more informative error types τ={1} and τ={2} for the four possible pairs of class indices $i_{1}$ and $i_{2}$ in Equations (A59) and (A60), since in this case $E_{s,\tau }^{i_{N}}$ in Equation (A64) is either directly an $E_{s}\left(\rho ,P_{\tau }\right)$ function or the straight-line tangent to it, in both cases shifted by a constant term given by $E_{s}^{i_{\tau^{c}}}\left(0,P_{\tau^{c}},\gamma_{\tau^{c}}\right)$.

Figure A1 shows the family of $E_{s,\tau }^{i_{N}}$ source functions respectively for independent and correlated sources, as a function of ρ where $P_{N}$ given by Equation (60) and τ={1}. For independent sources, we observe that the source functions $E_{s,\tau }^{1,1}$ and $E_{s,\tau }^{2,1}$ follow the solid blue line depicting $E_{s}\left(\rho ,P_{\tau }\right)$ as in Equation (A70) for a certain interval of ρ, and then take the tangent line beyond. A similar behavior is observed for the sources functions $E_{s,\tau }^{1,2}$ and $E_{s,\tau }^{2,2}$, which in this case follow or are tangent to the solid black line, the solid blue Gallager’s source function shifted by the constant function $E_{s}^{i_{\tau^{c}}}\left(0,P_{\tau^{c}},\gamma_{\tau^{c}}\right)$ as in Equation (A64).

For correlated sources, the source functions $E_{s,\tau }^{1,1}$ and $E_{s,\tau }^{2,1}$ follow the generalized Gallager’s source function given by Equation (A63) for a certain interval, but unlike independent sources they are not straight lines but a *curve* tangent to $E_{s,\tau }$ beyond that interval. Some intuition about this fact can be gained from the primal form of the source function $E_{s,\tau }^{i_{N}}$. Consider, for instance, the source function $E_{s,\tau }^{2,1}$ in Figure A1 for correlated sources, for which $i_{1}=2$ and $i_{2}=1$. The primal form of this source function $E_{s,\tau }^{2,1}$ can be obtained as a constrained optimization problem w. r. t. some auxiliary joint distribution ${\hat{P}}_{N}$. The interval in ρ where $E_{s,\tau }^{2,1}$ does not follow $E_{s,\tau }$ in the dual form (approximately for ρ≤0.5 in the figure) corresponds to the case where only one of the two constraints on the auxiliary distribution ${\hat{P}}_{N}$ is actually active in the primal form, where the constraint is given by $\sum_{u_{N}}{\hat{P}}_{N}\left(u_{N}\right)\log P_{\nu }\left(u_{\nu }\right)=\log\left(\gamma_{\nu }\right)$. This implies that, unlike the case of independent sources where each source has its auxiliary distribution ${\hat{P}}_{1}$ and ${\hat{P}}_{2}$ constrained, for correlated sources the joint auxiliary distribution ${\hat{P}}_{N}$ is not fully constrained but is the union of joint distributions with one constrained marginal distribution. This partial constraint manifests as a curve in ρ, rather than a straight line, in the dual form. A similar behavior is observed for $E_{s,\tau }^{1,2}$ and $E_{s,\tau }^{2,2}$, which instead of following the source function for joint source-channel coding in Equation (A63) for some intervals of ρ, they follow the curve

(A79) $$\begin{array}{c} \min_{\lambda_{\nu }\geq 0}\log\sum_{u_{\tau^{c}}}{\left(\sum_{u_{\tau }}P_{N}{\left(u_{N}\right)}^{\frac{1}{1+\rho }}{\left(\frac{P_{\nu }\left(u_{\nu }\right)}{\gamma_{\nu }}\right)}^{-\frac{{\left(-1\right)}^{i_{\nu }}\lambda_{\nu }}{1+\rho }}\right)}^{1+\rho }, \end{array}$$

corresponding to Equation (A62) when the constraint for one source is not active, i.e., ${\hat{\lambda }}_{\nu^{c}}=0$.
